# A genetic variant of the Wnt receptor LRP6 accelerates synapse degeneration during aging and in Alzheimer’s disease

**DOI:** 10.1126/sciadv.abo7421

**Published:** 2023-01-13

**Authors:** Megan E. Jones, Johanna Büchler, Tom Dufor, Ernest Palomer, Samuel Teo, Nuria Martin-Flores, Katharina Boroviak, Emmanouil Metzakopian, Alasdair Gibb, Patricia C. Salinas

**Affiliations:** ^1^ Department of Cell and Developmental Biology, University College London, London WC1E 6BT, UK.; ^2^ Wellcome Trust Sanger Institute, Wellcome Genome Campus, Hinxton, Cambridge CB10 1SA, UK.; ^3^ UK Dementia Research Institute, Department of Clinical Neuroscience, University of Cambridge, Cambridge CB2 0AH, UK.; ^4^ Department of Neuroscience, Physiology and Pharmacology, University College London, London WC1E 6BT, UK.

## Abstract

Synapse loss strongly correlates with cognitive decline in Alzheimer’s disease (AD), but the underlying mechanisms are poorly understood. Deficient Wnt signaling contributes to synapse dysfunction and loss in AD. Consistently, a variant of the *LRP6* receptor, (*LRP6-Val*), with reduced Wnt signaling, is linked to late-onset AD. However, the impact of *LRP6-Val* on the healthy and AD brain has not been examined. Knock-in mice, generated by gene editing, carrying this *Lrp6* variant develop normally. However, neurons from *Lrp6-val* mice do not respond to Wnt7a, a ligand that promotes synaptic assembly through the Frizzled-5 receptor. Wnt7a stimulates the formation of the low-density lipoprotein receptor-related protein 6 (LRP6)–Frizzled-5 complex but not if LRP6-Val is present. *Lrp6-val* mice exhibit structural and functional synaptic defects that become pronounced with age. *Lrp6-val* mice present exacerbated synapse loss around plaques when crossed to the *NL-G-F* AD model. Our findings uncover a previously unidentified role for *Lrp6-val* in synapse vulnerability during aging and AD.

## Teaser

Lrp6-val induces synapse loss in aging and in Alzheimer’s disease by inhibiting the formation of the Wnt receptor complex.

## INTRODUCTION

In Alzheimer’s disease (AD), memory impairment strongly correlates with synapse degeneration (*1*–*3*). Synaptic changes occur early in the disease, before amyloid-β (Aβ) plaque formation and neuronal loss (*3*, *4*). At the later stages of the disease, further synapse loss is observed around Aβ plaques (*5*–*8*). It is well documented that the accumulation of oligomeric forms of Aβ triggers synapse dysfunction and degeneration (*3*, *4*, *9*). Numerous studies demonstrate that Aβ oligomers interfere with signaling pathways, which are critical for maintaining synapse integrity, resulting in synapse weakening and loss (*10*, *11*). However, the molecular mechanisms that lead to synapse dysfunction and loss in AD remain poorly understood.

**Fig. 1.**
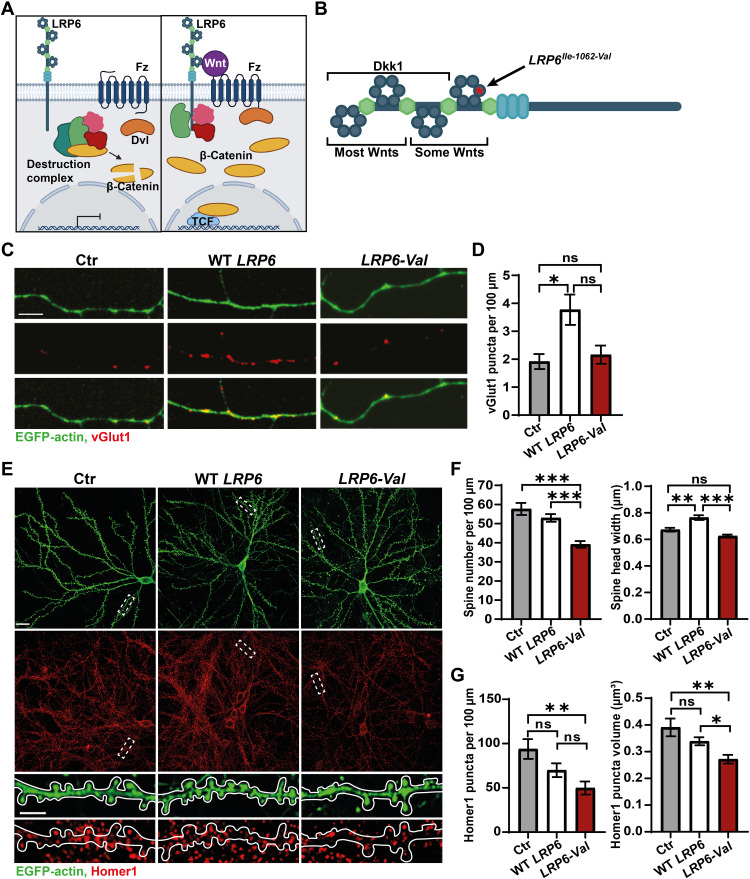
Expression of *LRP6-Val* induces synaptic defects in cultured neurons. (**A**) Diagram of canonical Wnt signaling. Left: In the absence of Wnt, β-catenin is sequestered and degraded by the destruction complex preventing transcription of Wnt target genes. Right: Wnt, LRP6, and Fz receptors form a complex. Activation of the pathway results in disheveled (Dvl) recruitment to the plasma membrane and disassembly of the destruction complex. β-Catenin accumulates and translocates to the nucleus enabling Wnt target gene transcription. (**B**) Schematic of LRP6 showing the location of the *Lrp6-val* SNP (red asterisk and arrow) and the areas to which the Wnt antagonist Dkk1 and Wnt ligands bind. (**C**) Confocal images of vGlut1 (red) puncta on isolated axons of neurons expressing enhanced green fluorescent protein (EGFP)–actin alone or EGFP-actin and human WT *LRP6* or human *LRP6-Val*. Scale bar, 5 μm. (**D**) WT *LRP6* promoted the assembly of presynaptic sites but *LRP6-Val* did not. *N* = 4 independent cultures, 10 to 12 axons per culture. Kruskal-Wallis with Dunn’s post hoc test. (**E**) Top: Confocal images of Homer1 (red) and GFP (green) of neurons expressing EGFP-actin and human WT *LRP6* or human *LRP6-Val*. Scale bar, 21 μm. Bottom: Higher-magnification images show dendritic spines (GFP; green) and Homer1 (red) puncta along dendrites. Scale bar, 5 μm. (**F**) Left: Expression of *LRP6-Val* reduced spine density. Three independent cultures, 8 to 10 cells per culture. One-way analysis of variance (ANOVA) with Tukey’s post hoc test. Right: Expression of *LRP6-Val* failed to increase spine size. *N* = 3 independent cultures, 8 to 10 cells per culture. Kruskal-Wallis with Dunn’s post hoc. (**G**) *LRP6-Val* expression led to smaller and fewer Homer1 puncta. *N* = 3 independent cultures, 8 to 10 images per culture. One-way ANOVA with Tukey’s post hoc test. Data are represented as means ± SEM. *P < 0.05, **P < 0.01, and ***P < 0.001. ns, not significant.

The canonical Wnt signaling pathway, required for synapse function and stability, is impaired in AD (Fig. 1A) (*12*–*14*). Wnt ligands and their receptors are expressed in many brain areas affected in AD (https://mouse.brain-map.org/). For instance, many Wnt ligands and Frizzled (Fz) receptors including Wnt7a, Wnt7b, and Fz5, and the Wnt co-receptor LRP6 are expressed in the postnatal and adult hippocampus (*13*, *15*–*18*). However, it is unclear whether these ligands and receptors act in an autocrine or paracrine fashion in neurons. Wnt ligands, their receptors, and co-receptors play a critical role in synaptogenesis during postnatal development (*18*–*21*) and in synapse integrity in the adult brain (*12*, *13*, *22*, *23*). The first piece of evidence that Wnt signaling is compromised in AD came from the finding that Dickkopf-1 (Dkk1), a secreted Wnt antagonist, is elevated in the brains of AD patients and AD mouse models (*24*, *25*). Notably, Dkk1 is required for Aβ-induced synapse degeneration as blockade of Dkk1 protects against Aβ-mediated synapse loss (*14*, *26*). Consistently, in vivo expression of Dkk1 in the adult brain induces synapse loss, long-term potentiation (LTP) defects (*12*), and memory impairment (*12*, *27*), as observed in AD mouse models. Second, conditional knockout (cKO) of *Lrp6* in an AD mouse model increases amyloid pathology and exacerbates cognitive deficits (*13*). Last, a genetic link between deficient Wnt signaling and AD came from the identification of three genetic variants of *LRP6* associated with late-onset AD (LOAD) (*28*, *29*). Notably, a nonsynonymous single-nucleotide polymorphism (SNP) (rs2302685) (Fig. 1B), which has an allele frequency of 0.17 in the European population (*30*), results in a conservative substitution of isoleucine to valine at amino acid 1062 (*LRP6^Ile-1062-Val^*; *LRP6-Val* herein). This substitution is located in the fourth β-propeller of the extracellular domain of LRP6 to which some Wnt ligands bind (Fig. 1B) (*31*–*33*). The *LRP6-Val* variant reduces Wnt signaling in cell lines in response to a Wnt ligand (*28*). However, the impact of this variant on brain development, neuronal connectivity, and amyloid pathology remains unexplored.

Here, we investigated the impact of the *Lrp6-val* variant on the adult and aging hippocampus and in the pathogenesis of AD by generating a novel knock-in (KI) mouse model using CRISPR-Cas9 genome editing. Homozygous *Lrp6-val* mice developed normally but showed structural and functional synaptic defects in the hippocampus that became more pronounced with age. In these mice, we observed decreased levels of canonical Wnt signaling with age. Notably, neurons from *Lrp6-val* mice were unable to respond to Wnt7a or Wnt3a to promote synapse formation. As Wnt ligands promote the interaction between LRP6 and Fz (*34*), we examined whether the LRP6-Val variant affects this interaction by focusing on Fz5, a Wnt7a receptor required for presynaptic assembly in hippocampal neurons (*15*). We found that Wnt7a increased the association between wild-type (WT) LRP6 and Fz5, whereas this interaction was significantly impaired in the presence of LRP6-Val. Consistently, expression of LRP6-Val reduced Wnt signaling. Next, we examined the contribution of the *Lrp6-val* variant to AD pathogenesis by crossing these mice to *hAPP^NL-G-F/NL-G-F^* (*NL-G-F*), a KI AD mouse model. The *Lrp6-val* variant significantly increased synapse degeneration in *NL-G-F* mice. The valine substitution in the extracellular domain of LRP6 impairs its Wnt7a-mediated interaction with Fz5, affecting downstream signaling. These findings represent a significant advancement in the Wnt and AD fields by linking a genetic variant that affects Wnt signaling with the pathogenesis of the disease.

## RESULTS

### *LRP6-Val* fails to stimulate synaptic assembly and induces spine loss

LRP6 is required for synapse formation and maintenance (*13*, *20*). Furthermore, the *LRP6-Val* variant is linked to LOAD (*28*), but its impact on neuronal connectivity has not been explored. To assess the effect of LRP6-Val on synapses, we expressed human WT *LRP6* or human *LRP6-Val* in cultured hippocampal neurons. Expression of WT *LRP6* increased the puncta number of vGlut1, a presynaptic marker, along axons (Fig. 1, C and D). In contrast, *LRP6-Val* failed to increase the number of presynaptic sites above control cells (Fig. 1, C and D). Thus, expression of the *LRP6-Val* variant is unable to induce presynaptic assembly in neurons.

We next examined whether the presynaptic assembly induced by expression of WT *LRP6* was ligand dependent as the function of this Wnt co-receptor requires Wnts for signaling (*35*). To block the synaptogenic activity on Wnts in hippocampal neurons, we used the secreted Fz-related protein 1 (sFRP1) (*19*). The addition of sFRP1 blocked the increase in the puncta number of Bassoon, a presynaptic marker, along axons induced by expression of WT *LRP6* (fig. S1). However, sFRP1 did not affect the phenotype of neurons expressing the *LRP6-Val* variant (fig. S1). These results indicate that endogenous Wnts are required for signaling through LRP6 resulting in presynaptic assembly.

We also examined the impact of LRP6-Val on dendritic spines (Fig. 1E). Expression of WT *LRP6* increased the spine head width but had no effect on spine density (Fig. 1, E and F). In contrast, expression of *LRP6-Val* failed to increase the spine head width and decreased the spine density when compared to control and WT *LRP6*-expressing cells (Fig. 1, E and F). Thus, *LRP6-Val* is unable to promote dendritic spine growth while inducing spine loss. No differences in puncta number or volume of Homer1, a postsynaptic marker, were found between control and WT *LRP6*-expressing neurons (Fig. 1, E and G). In contrast, consistent with the reduced number of spines, fewer Homer1 puncta and a decrease in puncta volume were observed in *LRP6-Val*–expressing neurons compared to control neurons (Fig. 1, E and G). Thus, expression of *LRP6-Val* in neurons decreases the formation of both pre- and postsynaptic sites compared to WT *LRP6*.

### *Lrp6-val* KI mice develop normally, and LRP6-Val does not affect its synaptic localization

**Fig. 2.**
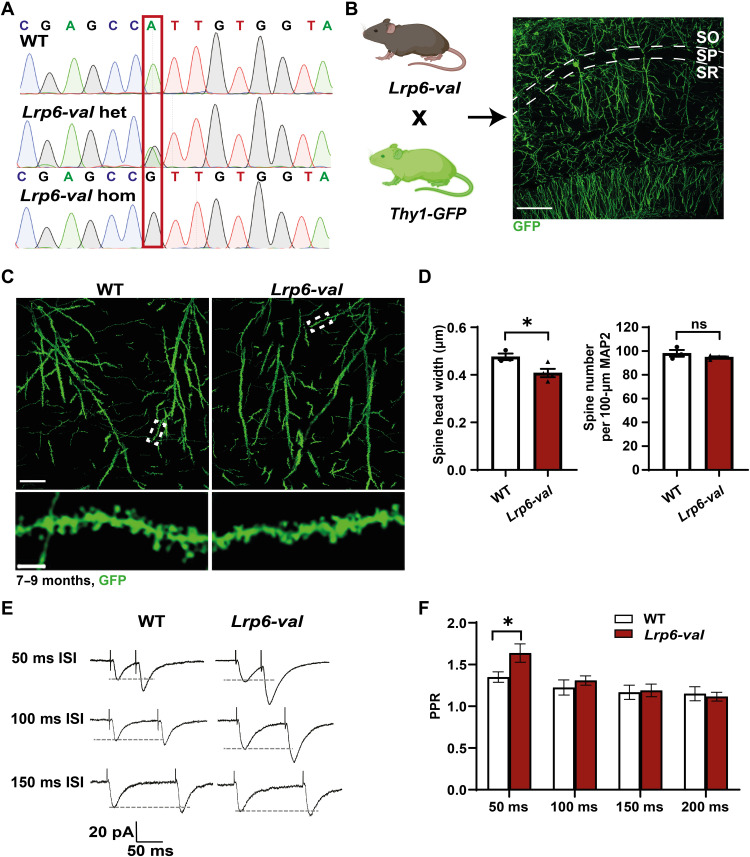
*Lrp6-val* mice exhibit synaptic defects at 7 to 9 months. (**A**) Sanger trace examples of WT, *Lrp6-val* heterozygous (*Lrp6-val* het), and *Lrp6-val* homozygous (*Lrp6-val* hom) KI mice. (**B**) Dendritic spines were analyzed in *Lrp6-val* hom KI mice crossed to a *Thy1-GFP* line. SO, stratum oriens; SP, stratum pyramidale; SR, stratum radiatum. Scale bar, 100 μm. (**C**) Confocal images of apical dendrites of CA1 pyramidal neurons of WT and *Lrp6-val* mice at 7 to 9 months. Scale bar, 25 μm. Insets show spines along a dendrite. Scale bar, 3 μm. (**D**) *Lrp6-val* mice display reduced spine head width. WT, *N* = 3; *Lrp6-val N* = 4. Unpaired *t* test. *P < 0.05. (**E**) Representative paired-pulse recordings of synaptic currents from WT and *Lrp6-val* brain slices at different ISIs. (**F**) Graph displays the mean PPR from all recorded cells. *Lrp6-val* increased the PPR at 50-ms ISIs. *N =* 11 to 21 cells recorded from four to five animals per genotype. Repeated measures one-way ANOVA with Tukey’s post hoc test. *P < 0.05. Data are represented as means ± SEM.

To investigate the in vivo role of the *Lrp6-val* variant, we generated a novel KI mouse model using CRISPR-Cas9 genome editing. The *Lrp6* A→G point mutation, which results in the substitution of isoleucine for valine, was introduced at the endogenous *Lrp6* locus in the mouse genome, creating a mouse line that carries the *Lrp6-val* variant globally. DNA sequencing confirmed the successful generation of both heterozygous (*Lrp6-val* het) and homozygous (*Lrp6-val* hom) KI animals (Fig. 2A). *Lrp6-val* hom mice developed normally with no visible morphological abnormalities or changes in weight (fig. S2, A and B). Furthermore, *Lrp6-val* het and *Lrp6-val* hom mice exhibited similar synaptic phenotypes (see below). Here, we primarily focused our attention on *Lrp6-val* hom (*Lrp6-val*) mice.

We next investigated whether the *Lrp6-val* variant was differentially expressed or affected its synaptic localization. The mRNA and protein levels of LRP6 in the hippocampus of *Lrp6-val* mice were unchanged (fig. S2, C to E). To assess its synaptic localization, we used structured illumination microscopy (SIM). Both WT LRP6 and LRP6-Val were present at approximately 80% of excitatory synapses (fig. S2, F and G), but no differences in LRP6 localization were observed between neurons from WT and *Lrp6-val* mice (fig. S2G). Thus, carrying the *Lrp6-val* variant does not alter the levels or the synaptic localization of this co-receptor.

### *Lrp6-val* causes progressive synaptic defects with age

Spine formation and growth are modulated by Wnt signaling in the hippocampus (*18*, *19*, *23*, *36*). Given that the expression of *LRP6-Val* failed to stimulate spine growth and induced spine loss in cultured neurons (Fig. 1, E and F), we examined the in vivo impact of *Lrp6-val* on these postsynaptic structures. We analyzed the dendritic spines on the apical dendrites of Cornu Ammonis-1 (CA1) pyramidal neurons in adult *Lrp6-val* KI mice crossed to a *Thy1-GFP* expressing line (Fig. 2B) (*37*). Although no differences in the spine density were observed, the spine head width was reduced in *Lrp6-val* mice compared to WT mice at 7 to 9 months (Fig. 2, C and D). Thus, adult mice carrying the *Lrp6-val* variant display impaired spine growth.

Given our data on the synaptic localization of LRP6 and the role of Wnt signaling in synaptic function (*38*, *39*), we examined synaptic transmission in the *Lrp6-val* mice at Schaffer collateral (SC)–CA1 synapses, where deficient Wnt signaling leads to defects in synaptic transmission (*12*). Evoked excitatory postsynaptic currents (EPSCs), in response to SC stimulation of increasing intensity [input-output (I/O) curve], were recorded in CA1 pyramidal cells at 7 to 9 months. No significant differences were observed between WT and *Lrp6-val* mice even at high-stimulation intensities (fig. S3, A and B), suggesting that basal synaptic transmission at SC-CA1 synapses is unaffected by the presence of the *Lrp6-val* variant at this age.

Wnt signaling–deficient mice exhibit reduced neurotransmitter release probability (*22*, *40*). We therefore investigated the possible defects in neurotransmitter release. EPSCs evoked at brief intervals at SC-CA1 synapses were recorded from WT and *Lrp6-val* mice at 7 to 9 months. We analyzed the paired-pulse ratio (PPR), which depends on presynaptic short-term plasticity mechanisms and is inversely correlated with release probability (*41*, *42*). *Lrp6-val* mice exhibited increased PPRs compared to WT mice at 50-ms interstimulus intervals (ISIs), consistent with a reduced release probability at 7 to 9 months (Fig. 2, E and F). Thus, neurotransmitter release is compromised in adult *Lrp6-val* mice.

**Fig. 3.**
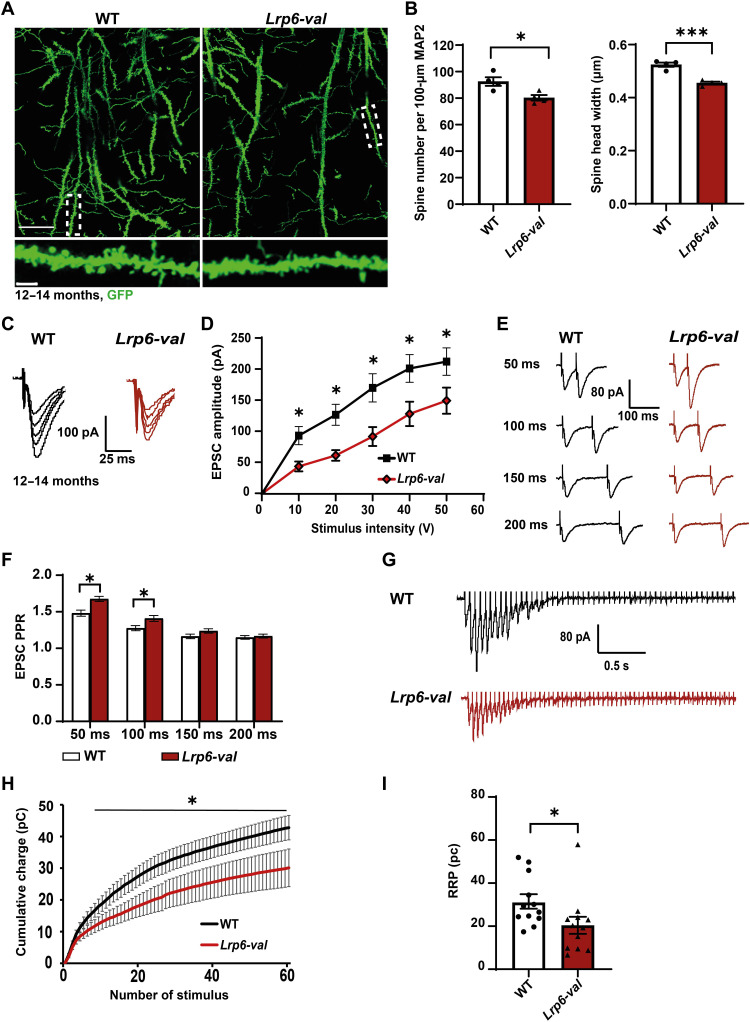
*Lrp6-val* mice display postsynaptic defects and impaired basal synaptic transmission, synaptic vesicle release, and RRP size at 12 months. (**A**) Top: Dendritic spines were analyzed at 12 to 14 months in *Lrp6-val* KI mice crossed to a *Thy1-GFP* line. Scale bar, 25 μm. Bottom: Confocal images of regions of interest containing apical dendrites of CA1 pyramidal neurons in WT and *Lrp6-val* mice. Scale bar, 3 μm. (**B**) *Lrp6-val* mice have smaller and fewer spines. WT, *N* = 4; *Lrp6-val*, *N* = 4. Unpaired *t* test. (**C**) Representative traces of EPSCs elicited at increasing stimulation voltages with an average of three responses for each stimulus voltage. (**D**) I/O curves showing a significant reduction in EPSC amplitude in hippocampal slices from *Lrp6-val* mice. *N* = 12 to 13 cells from four animals per genotype. Repeated measure one-way ANOVA with Tukey’s post hoc test. (**E**) Representative traces of paired pulse evoked EPSCs at different ISIs using brain slices from WT and *Lrp6-val* mice. (**F**) Graph displays the mean PPR from all cells. *Lrp6-val* mice display increased PPR at 50- and 100-ms ISI. *N* = 13 to 14 cells from four animals per genotype. Repeated measure one-way ANOVA with Tukey’s post hoc test. (**G**) Representative traces of EPSCs elicited by a 20-Hz electrical stimulation for 3 s recorded from WT and *Lrp6-val* mice. (**H**) Graph showing reduced mean cumulative charge in *Lrp6-val* mice. *N* = 12 cells from four animals per genotype. Repeated measure one-way ANOVA with Tukey’s post hoc test. (**I**) Graph displays the RRP size, obtained from all cells. *Lrp6-val* mice exhibited a reduced RRP. *N* = 12 cells from four animals per genotype. Unpaired Student’s *t t*est. Data are represented as means ± SEM. *P < 0.05 and ***P < 0.001.

Given that cKO mice for *Lrp6* exhibit age-dependent synaptic deficits (*13*), we interrogated whether synaptic defects become more pronounced with age. We evaluated dendritic spines in older *Lrp6-val;Thy1-GFP* mice (Fig. 3A). Our analyses revealed a significant decrease in both spine density and head width in *Lrp6-val* mice compared to WT mice at 12 to 14 months (Fig. 3B). As the spine size but not spine number was affected in 7- to 9-month-old *Lrp6-val* mice, these results demonstrate that spine deficits become more severe with age.

Next, we evaluated basal synaptic transmission in 12-month-old mice by performing I/O curve recordings in hippocampal slices. The amplitude of evoked EPSCs was significantly smaller in 12-month-old *Lrp6-val* mice compared to WT mice at all stimulus intensities (Fig. 3, C and D). Thus, the *Lrp6-val* variant impairs basal synaptic transmission at this age but not at 7 to 9 months (fig. S3, A and B). We then measured neurotransmitter release probability and found that the PPR was significantly higher in *Lrp6-val* mice compared to WT mice at both 50- and 100-ms ISI, consistent with a reduction in release probability (Fig. 3, E and F). Given that PPR was significantly higher at 50-ms but not at 100-ms ISI in *Lrp6-val* mice at 7 to 9 months, these results suggest that defects in neurotransmitter release are more pronounced in older animals.

To further define the role of *Lrp6-val* in neurotransmitter release at 12 months, we recorded responses to a 3-s high-frequency stimulus train (20 Hz), which fully depletes presynaptic terminals of the readily releasable pool (RRP) (*43*). Using the first-order correction for vesicle recycling (*43*), a significant reduction in the size of the RRP was observed in 12-month-old *Lrp6-val* mice when compared to WT mice (Fig. 3, G to I). However, synaptic vesicle fusion efficiency and recycling rate were unaffected (fig. S3C), suggesting that the defect in release probability is due to a reduced RRP.

**Fig. 4.**
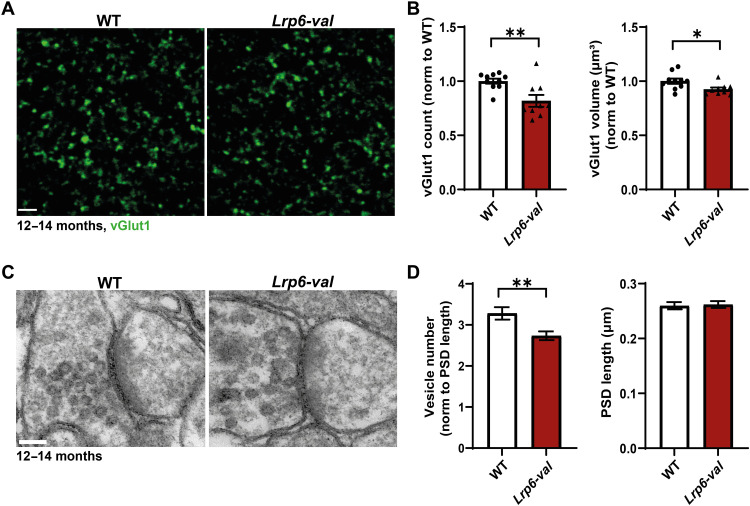
Presynaptic defects of *Lrp6-val* mice at 12 to 14 months. (**A**) Confocal images of vGlut1-labeled excitatory presynaptic terminals in the CA1 SR area of WT and *Lrp6-val* mice at 12 to 14 months. Scale bar, 2 μm. (**B**) *Lrp6-val* mice had fewer and smaller vGlut1 puncta. WT, *N* = 10; *Lrp6-val*, *N* = 9. Unpaired *t* test. *P < 0.05 and **P < 0.01. (**C**) EM images of an excitatory synapse of 12- to 14-month-old WT and *Lrp6-val* mice. Scale bar, 100 nm. (**D**) *Lrp6-val* mice had fewer synaptic vesicles, but no changes in PSD length were observed. *N* = 5, 19 to 25 images per animal. Mann-Whitney test. **P < 0.01. Data are represented as means ± SEM.

The defects in vesicular release probability at 12 months suggested potential structural changes at presynaptic terminals of *Lrp6-val* mice. To investigate this hypothesis, we evaluated the number and size of presynaptic boutons using the presynaptic marker, vGlut1, in the CA1 stratum radiatum (SR) of *Lrp6-val* mice at 12 to 14 months (Fig. 4A). *Lrp6-val* mice had smaller and fewer vGlut1 puncta compared to WT mice (Fig. 4B). As *Lrp6-val* mice exhibited deficits in synaptic vesicle release, due to a smaller RRP (Fig. 3), we assessed possible ultrastructural changes by electron microscopy (EM). We observed fewer synaptic vesicles at presynaptic terminals but no differences in the length of the postsynaptic density (PSD) at SC-CA1 synapses of *Lrp6-val* mice (Fig. 4, C and D). These findings indicate that impaired neurotransmitter release in *Lrp6-val* mice is due to a reduction in synaptic vesicle number, consistent with a reduced RRP.

**Fig. 5.**
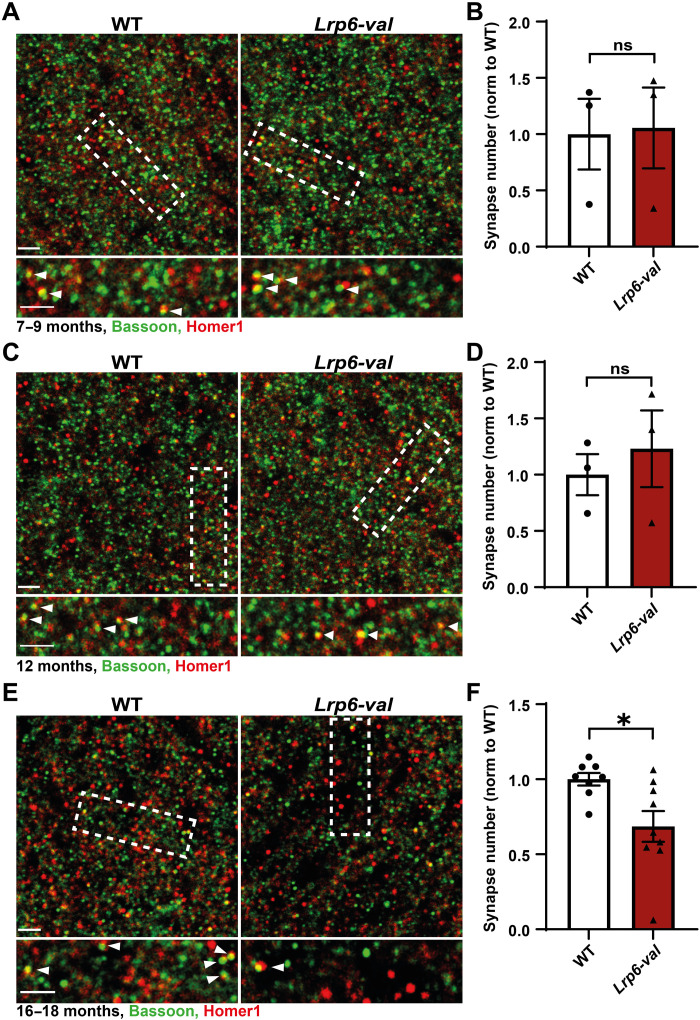
*Lrp6-val* mice exhibit synapse loss with age. (**A**, **C**, and **E**) Confocal images of the CA1 SR of WT and *Lrp6-val* mice labeled with Bassoon (green) and Homer1 (red) at 7 to 9 months (A), 12 months (C), and 16 to 18 months (E). Scale bars, 2.5 μm. Insets display high-magnification images of synapses. Scale bars, 2 μm. (**B** and **D**) Quantification of synapse number, based on the colocalization of pre- and postsynaptic puncta, showed no differences between WT and *Lrp6-val* mice at 7 to 9 months (B) or 12 months (D). *N* = 3 per genotype. Unpaired *t* test. (**F**) Synapse number was significantly reduced in *Lrp6-val* mice 16 to 18 months. WT, *N* = 8; *Lrp6-val*, *N* = 9. Unpaired *t* test. *P < 0.05. Data are represented as means ± SEM.

Given that defects at the pre- and postsynaptic sites were exacerbated with age in *Lrp6-val* mice, we compared the number of excitatory synapses at the different ages. Synapses were quantified on the basis of the colocalization of Bassoon and Homer1, pre- and postsynaptic markers, respectively, in the CA1 SR region. At 7 to 9 months and 12 months, no differences were observed between WT and *Lrp6-val* mice (Fig. 5, A to D). However, a significant reduction in the number of excitatory synapses was detected at 16 to 18 months in *Lrp6-val* mice when compared to WT mice (Fig. 5, E and F). Because of the age of these animals, we examined possible neuronal loss, which could affect synapse number, in the stratum pyramidale layer using 4′,6-diamidino-2-phenylindole (DAPI) and NeuN (fig. S4A). No difference in the percentage of NeuN-positive cells was identified between WT and *Lrp6-val* mice at 16 to 18 months (fig. S4B). Thus, the *Lrp6-val* variant confers increased synaptic vulnerability as animals age, a process that is not due to changes in neuronal number.

As the *Lrp6-val* variant is predominantly present as heterozygous in the human population (*30*), we also analyzed heterozygous *Lrp6-val* mice at 12 to 14 months. Although no differences were detected in the presynaptic marker vGlut1 between WT and heterozygous mice, homozygous mice exhibited a reduction in vGlut1 puncta number (fig. S5A). Postsynaptically, both heterozygous and homozygous *Lrp6-val* mice exhibited a decrease in the number and size of dendritic spines when compared to WT mice (fig. S5B). Thus, carrying one allele of the *Lrp6-val* confers postsynaptic vulnerability.

### Wnt signaling is impaired in *Lrp6-val* mice, and *Lrp6-val* neurons do not respond to Wnt7a

To start addressing the molecular mechanisms through which aged *Lrp6-val* mice exhibit synaptic defects, we investigated whether canonical Wnt signaling was affected in aged mice. We therefore evaluated the mRNA levels of *Axin2*, a target of canonical Wnt signaling (fig. S6A) (*44*). *Axin2* expression was not affected in 4- to 7-month-old *Lrp6-val* mice (fig. S6B), but it was significantly decreased in 12- to 15-month-old *Lrp6-val* mice compared to WT (fig. S6C). Thus, Wnt signaling is compromised with age in *Lrp6-val* mice, consistent with the appearance of synapse defects.

**Fig. 6.**
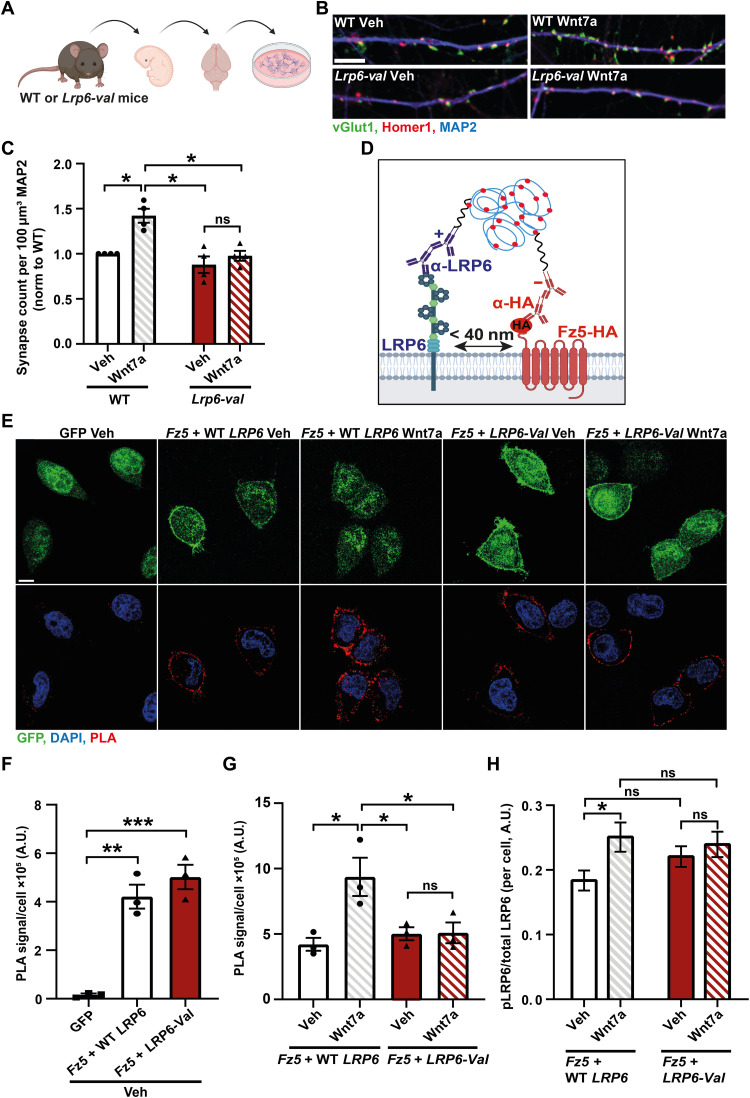
Neurons from *Lrp6-val* mice fail to respond to Wnt7a and the presence of *LRP6-Val* affects the formation of the Wnt receptor complex and downstream signaling. (**A**) Diagram depicting hippocampal neuron isolation from WT and *Lrp6-val* mice. (**B**) Images of WT and *Lrp6-val* neurons treated with recombinant Wnt7a. vGlut1 (green), Homer1 (red), and MAP2 (Blue). Scale bar, 5 μm. (**C**) Wnt7a (200 ng/ml) increased synapse number in WT neurons but not in *Lrp6-val* neurons. *N* = 4 independent cultures. Two-way ANOVA with Games-Howell post hoc test. (**D**) Schematic of proximity ligation assay (PLA) to detect LRP6 and Fz5-HA interaction in close proximity (<40 nm). (**E**) Confocal images of HeLa cells expressing GFP (control), *Fz5-HA*, and WT *LRP6* or *LRP6-Val* treated with control vehicle (Veh) or Wnt7a. GFP, green; PLA, red; and DAPI, blue. Scale bar, 10 μm. (**F**) The PLA signal intensity per cell was increased in cells expressing *Fz5-HA* and WT *LRP6* or *LRP6-Val* compared to cells expressing GFP. *N* = 3 independent experiments. One-way ANOVA with Tukey’s post hoc test. (**G**) Wnt7a increased the PLA signal in cells expressing WT *LRP6* and *Fz5-HA* but not in cells expressing *LRP6-Val* and *Fz5-HA*. *N* = 3 independent experiments. Two-way ANOVA with Tukey’s post hoc test. (**H**) Wnt7a increased pLRP6 when normalized to total LRP6 in HeLa cells expressing WT *LRP6* and *Fz5-HA* but not in cells expressing *LRP6-Val* and *Fz5-HA*. WT *LRP6* + *Fz5-HA* Veh, *N* = 50 cells; WT *LRP6* + *Fz5-HA* Wnt7a, *N* = 53 cells; *LRP6-Val* + *Fz5-HA* Veh, *N* = 71 cells; and *LRP6-Val* + *Fz5-HA* Wnt7a, *N* = 61 cells from three independent experiments. Kruskal-Wallis with Dunn’s post hoc test. Data are represented as means ± SEM. *P < 0.05, **P < 0.01, and ***P < 0.001. A.U. arbitrary units.

The above findings led us to hypothesize that the LRP6-Val receptor does not signal properly. We therefore interrogated whether neurons from *Lrp6-val* mice responded to exogenous Wnts (Fig. 6A). Previous studies demonstrate that Wnt7a promotes the formation of excitatory synapses in hippocampal neurons (*15*–*18*). Consistently, Wnt7a significantly increased the number of synapses in neurons from WT mice compared to neurons exposed to a control vehicle (Fig. 6, B and C). However, Wnt7a was unable to increase synapse number in hippocampal neurons from *Lrp6-val* mice (Fig. 6B and C). Crucially, the same effect was observed with a second concentration of Wnt7a (fig. S7, A and B). We also examined whether neurons from *Lrp6-val* mice respond to Wnt3a, another ligand involved in presynaptic assembly (*16*, *45*). Moreover, the Ile to Val amino acid substitution in LRP6 is in the Wnt3a binding site (*31*–*33*), and expression of *LRP6-Val* in a cell line reduces Wnt3a-mediated signaling (*28*). We found that Wnt3a increased the number of synapses in WT neurons but failed to increase synapse number in *Lrp6-val* neurons (fig. S7, A and C). Thus, *Lrp6-val* neurons respond to neither Wnt7a nor Wnt3a. The lack of response to Wnt ligands was not due to decreased levels of the LRP6-Val receptor at the plasma membrane, as the surface levels of LRP6 were the same between WT and *Lrp6-val* neurons (fig. S7, A, D, and E). Thus, *Lrp6-val* impairs the ability of neurons to respond to exogenous Wnt ligands without affecting its surface localization.

### LRP6-Val impairs the formation of the Wnt7a-induced LRP6-Fz5 complex and downstream signaling

The lack of response to Wnt7a and Wnt3a in neurons from *Lrp6-val* mice suggested that the presence of LRP6-Val could impair the formation of a complex between LRP6 and Fz receptors, which is crucial for the activation of the canonical Wnt signaling pathway (*34*). We specifically chose to examine the interaction between Fz5 and LRP6 because this Fz receptor is required for Wnt7a-mediated presynaptic assembly in hippocampal neurons (*15*). We coexpressed *Fz5-HA* with WT *LRP6* or *LRP6-Val* in HeLa cells to evaluate their interaction, which is induced by Wnt7a, using proximity ligation assay (PLA), a technique that allows the detection of protein-protein interactions at less than 40 nm in cells (Fig. 6D) (*46*, *47*). The PLA signal was elevated in cells expressing *Fz5-HA* and WT *LRP6* or *LRP6-Val* when compared to control green fluorescent protein (GFP)–expressing cells (Fig. 6, E and F). No differences in the interaction were observed between cells expressing *Fz5-HA* and WT *LRP6* or *LRP6-Val* under basal conditions (Fig. 6, E to G). In contrast, Wnt7a significantly increased the PLA signal intensity in cells expressing *Fz5-HA* and WT *LRP6* but not in cells expressing *Fz5-HA* and *LRP6-Val* (Fig. 6, E and G). These results were not due to changes in the surface levels of these receptors, as determined by surface biotinylation (fig. S8, A to E). Together, these results demonstrate that the presence of LRP6-Val impairs the formation of the Wnt-induced LRP6-Fz receptor complex, which is required for signaling.

We next examined whether downstream signaling was affected by the presence of LRP6-Val. The formation of the LRP6-Fz complex promotes the intracellular phosphorylation of LRP6 at multiple sites, including at serine-1490, which is important for LRP6 function (*48*). We therefore tested whether this posttranslational modification was affected by the LRP6-Val variant. Cells expressing WT *LRP6* and *Fz5-HA* or *LRP6-Val* and *Fz5-HA* were exposed to recombinant Wnt7a, and the level of phosphorylated LRP6 (pLRP6) at serine-1490 was evaluated by immunofluorescence microscopy. As expected, Wnt7a significantly increased the level of pLRP6 in cells expressing WT *LRP6* (Fig. 6H and fig. S8F). However, no changes were observed in the pLRP6 levels in cells expressing the *LRP6-Val* variant (Fig. 6H and fig. S8F). Thus, the presence of LRP6-Val affects the formation of the LRP6-Fz5 complex and subsequent downstream signaling.

### *Lrp6-val* does not affect plaque load in *NL-G-F* mice

The findings that the *LRP6-Val* variant is associated with LOAD (*28*) and that cKO of *Lrp6* in neurons of the APP/PS1 AD mouse model exacerbates the formation of Aβ plaques (*13*) led us to interrogate the impact of the *Lrp6-val* variant on amyloid pathology. We crossed the *Lrp6-val* mice to the *NL-G-F*, a KI AD mouse model that carries a humanized Aβ region of APP with three mutations associated with AD (*49*). In *NL-G-F* mice, plaque deposition begins around 2 months, with a significant increase in the number at 7 months (*49*). We assessed the impact of *Lrp6-val* on plaque load in homozygous *NL-G-F* mice. No differences in plaque burden were detected between *NL-G-F* and *NL-G-F;Lrp6-val* mice at 2, 7, and 10 months (fig. S9, A to F). Furthermore, Aβ coverage (area covered by Aβ) was unaltered in *NL-G-F;Lrp6-val* mice compared to *NL-G-F* mice at 7 and 10 months (fig. S9, C to F). Thus, the presence of the *Lrp6-val* variant does not exacerbate plaque load in *NL-G-F* mice at the ages examined.

Next, we examined the levels of soluble and insoluble Aβ42, as this peptide is the most abundant Aβ species in *NL-G-F* mice (*49*). However, no differences in the level of Aβ42 was observed between *NL-G-F* and *NL-G-F;Lrp6-val* mice at 10 months (fig. S10). These results are consistent with our findings that Aβ plaque number and Aβ coverage are unaffected in *NL-G-F* mice when carrying the *Lrp6-val* variant.

### *Lrp6-val* exacerbates synapse loss in *NL-G-F* mice

Although the impact of *Lrp6* cKO on Aβ plaque load has been examined in the context of AD (*13*), changes in synapse density were not investigated. Our findings that *Lrp6-val* mice exhibit synaptic defects led us to interrogate the contribution of this SNP to synapse vulnerability in AD. We therefore evaluated the impact of *Lrp6-val* on synapses in *NL-G-F* mice at 2 months of age, when plaques begin to form (*49*). However, no differences in synapse number were observed between WT, *Lrp6-val*, *NL-G-F*, and *NL-G-F;Lrp6-val* mice at this early stage (fig. S11, A and B).

**Fig. 7.**
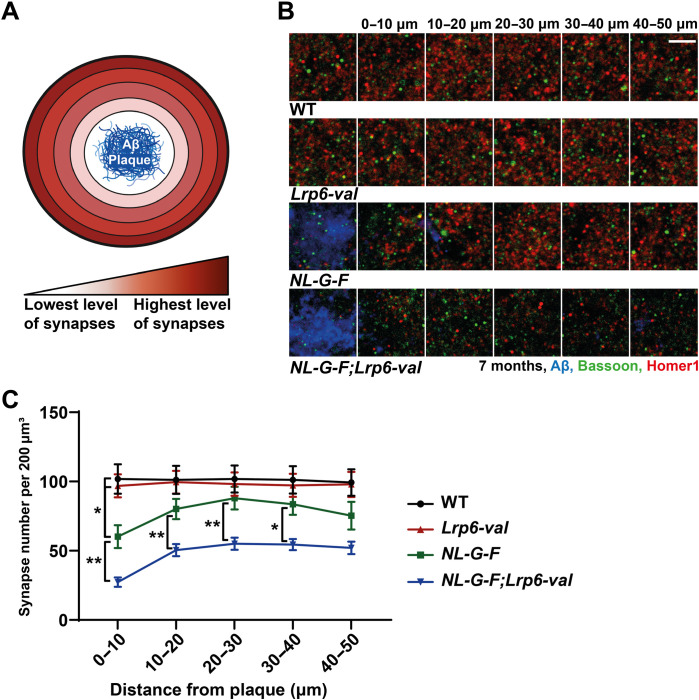
*Lrp6-val* exacerbates synapse loss around plaques in NL-G-F mice at 7 months. (**A**) Diagram shows synapse loss around an Aβ plaque (blue). (**B**) Confocal images of Bassoon (green) and Homer1 (red) at increasing distances from the center of an Aβ plaque (blue) in *NL-G-F* and *NL-G-F;Lrp6-val* mice or an equivalent point in WT and *Lrp6-val* mice, in the CA1 SR at 7 months. Scale bar, 4 μm. (**C**) *NL-G-F* mice displayed fewer synapses compared to WT mice or to *Lrp6-val* mice at 0 to 10 μm from the center of a plaque. Synapse number was reduced in *NL-G-F;Lrp6-val*miceat 0 to 40 μm from the center of a plaque compared to *NL-G-F* mice. A significant reduction in synapse number was detected in *NL-G-F;Lrp6-val* micewhen compared to WT mice or *Lrp6-val* mice at all distances from the center of a plaque. WT = 17 slices from six animals, *Lrp6-val* = 18 slices from six animals, *NL-G-F* = 18 slices from seven animals, and *NL-G-F;Lrp6-val* = 19 slices from seven animals. Repeated measure two-way ANOVA with Tukey’s post hoc test. *P < 0.05 and **P < 0.01. Data are represented as means ± SEM.

We next investigated the impact of the *Lrp6-val* variant on synapses at 7 months of age in *NL-G-F* mice, by which time a significant number of plaques are present (*49*). Given that synapse loss is particularly pronounced around Aβ plaques (Fig. 7A) (*5*–*8*), synapse number was quantified at increasing distances from the center of a plaque or from a similar area in WT and *Lrp6-val* mice, in the CA1 SR (Fig. 7B). At 0 to 10 μm from the center of a plaque, a significant reduction in synapse number was observed in *NL-G-F* mice when compared to WT mice or to *Lrp6-val* mice (Fig. 7, B and C). A further decrease in synapse number was observed between *NL-G-F;Lrp6-val* double-mutant mice when compared to *NL-G-F*, *Lrp6-val*, or WT mice. This effect was also observed at further distances from the center of a plaque (Fig. 7, B and C). Thus, carrying the *Lrp6-val* variant exacerbates synapse loss around plaques in *NL-G-F* mice.

To determine whether the enhanced synapse degeneration at 7 months was due to deregulation of Wnt signaling components, we examined the expression of key Wnt ligands and receptors by quantitative polymerase chain reaction (qPCR) in the hippocampi of WT, *Lrp6-val*, *NL-G-F*, and *NL-G-F;Lrp6-val* mice. However, no differences were detected in the expression of the Wnt receptors *Lrp6* and *Fz5* or the Wnt ligands *Wnt7a* and *Wnt7b* (fig. S12).

We also examined whether synapse number was more pronounced in the double-mutant mice when compared to *NL-G-F* mice at 10 months as Aβ pathology becomes more severe with age in *NL-G-F* mice (*49*). We found no significant differences in synapse loss around plaques between *NL-G-F* and *NL-G-F; Lrp6-val* mice at this age (fig. S11, C and D). Together, our results demonstrate that carrying the LRP6-Val receptor accelerates synapse loss in *NL-G-F* mice when synaptic defects become evident but not with progressive pathogenesis.

## DISCUSSION

Here, we evaluate the impact of the *LRP6-Val* variant, which is linked to LOAD, on synaptic connectivity during aging and in AD. We generated a novel KI mouse model carrying this variant. Analyses of homozygous *Lrp6-val* mice reveal that this SNP induces age-associated defects in synaptic transmission and neurotransmitter release. *Lrp6-val* mice also exhibit progressive structural synaptic defects in the hippocampus. The presence of *Lrp6-val* exacerbates synapse loss around plaques in the *NL-G-F* AD model. Investigation into the molecular mechanisms underlying the synaptic defects elicited by LRP6-Val reveals a defect in the formation of the LRP6-Fz5 complex mediated by Wnt7a, which explains the decreased downstream signaling that is only evident with age. Thus, our studies also reveal a previously unrecognized molecular mechanism by which this variant affects Wnt signaling and uncover a role for *LRP6-Val* in synapse degeneration in AD.

The LRP6 receptor, which localizes to synapses, promotes the assembly of synapses. Using superresolution microscopy, we found that LRP6 is present at both pre- and postsynaptic sites. Expression of WT *LRP6* in neurons increases the number of presynaptic puncta and promotes spine growth, consistent with activation of the Wnt pathway in neurons (*16*, *18*) and in agreement with a previous study showing that LRP6 localizes to both pre- and postsynaptic sites and is required for synaptogenesis in cultured neurons (*20*). In contrast, expression of the *LRP6-Val* variant in neurons neither increases the number of presynaptic sites nor affects spine size but induces spine loss. Together, these findings demonstrate that expression of the *LRP6-Val* variant in hippocampal neurons fails to promote synapse formation.

Homozygous *Lrp6-val* mice exhibit structural and functional synaptic defects that become more pronounced with age. At 7 to 9 months, we observe reduced spine head width without changes in spine number or synapse number, whereas at 12 to 14 months both spine number and head width and the number of vGlut1 puncta are decreased. Moreover, defects in basal synaptic transmission and synaptic vesicle release, due to a smaller RRP, are exacerbated from 7 to 9 months to 12 to 14 months in homozygous *Lrp6-val* mice. However, no changes in synapse number are observed at these ages. In contrast, homozygous *Lrp6-val* mice exhibit a reduced number of excitatory synapses at 16 to 18 months. Heterozygous *Lrp6-val* mice also exhibit synaptic defects, suggesting that carrying a single allele of this variant confers synaptic vulnerability. Thus, the presence of the LRP6-Val variant contributes to progressive synaptic dysfunction and synapse degeneration.

Multiple variants of *LRP6* have been linked to various age-associated diseases (*28*, *50*, *51*), including the *LRP6-Val* variant studied in this work, which has an allele frequency of 0.17 in the European population (*30*). First, *LRP6-Val* is associated with a 60% increase in bone fracture risk in older men (*50*). Second, *LRP6-Val* is a risk factor for carotid artery atherosclerosis in hypertensive patients over the age of 65 (*51*). Last, *LRP6-Val* is associated with LOAD (*28*). Thus, carrying the *LRP6-Val* variant results in age-related phenotypes that extend beyond the nervous system.

What are the molecular mechanisms that contribute to synaptic defects in the *Lrp6-val* mice? The synaptic deficits of *Lrp6-val* mice are not due to defects in the levels or localization of the LRP6-Val protein, as similar protein levels are found at synapses and the cell surface when compared to wildtype LRP6. This finding suggests possible defects in downstream signaling. Consistent with this hypothesis, Wnt7a and Wnt3a fail to induce excitatory synapse formation in neurons isolated from *Lrp6-val* mice. This lack of response correlates with defects in the interaction between Fz5 and LRP6-Val in response to Wnt7a and reduced downstream Wnt signaling as determined by the levels of pLRP6 and the levels of *Axin2* expression at 12 to 15 months in *Lrp6-val* mice. Moreover, expression of *LRP6-Val* attenuates canonical Wnt signaling as evaluated by the TOPFlash assay (*28*). Thus, the presence of LRP6-Val impairs the formation of the Wnt receptor complex in response to Wnt7a, which decreases downstream signaling resulting in synaptic dysfunction and synapse degeneration.

The lack of response of *Lrp6-val* neurons to exogenous Wnt7a or Wnt3a suggests that LRP6-Val could act like a null mutant. However, *Lrp6-val* mice do not display developmental defects as observed in *Lrp6* full KO mice, which die at birth due to severe embryonic defects (*52*). Thus, the *Lrp6-val* exhibits a hypomorphic phenotype. The lack of an early embryonic phenotype in the *Lrp6-val* mice could be due to the differential expression of different auxiliary proteins for the LRP6 co-receptor at different ages. These auxiliary proteins could compensate for signaling defects in the *Lrp6-val* mice during development but not in the adult brain.

The synaptic defects are manifested with age in the *Lrp6-val* mice. A possible explanation for this phenotype is that canonical Wnt signaling is dampened with age. Canonical Wnt signaling and several Wnt ligands, including Wnt7a, are reduced in the aging brain (*53*, *54*). On the basis of these findings, we propose that reduced levels of Wnt proteins with age combined with the presence of a less functional receptor, such as LRP6-Val, contributes to the manifestation of age-dependent synapse loss in *Lrp6-val* mice.

In the context of AD, our studies demonstrate that carrying the *Lrp6-val* variant increases synapse vulnerability. *Lrp6-val* exacerbates synapse loss surrounding plaques in the *NL-G-F* model at 7 months. This is not due to differential expression of Wnt components, increased Aβ42 levels, or plaque load. Our findings are in contrast with those reported using the cKO of *Lrp6* crossed to the APP/PS1 AD model, which exhibit increased Aβ40 and Aβ42 levels and enhanced plaque load (*13*). These differing results could be due to various reasons. First, our KI model contains a single–amino acid substitution in the *Lrp6* gene, which is likely to result in a milder phenotype than that observed in the cKO model. Second, the cKO of *Lrp6* was studied in APP/PS1, a transgenic model that overexpresses mutant APP and Presenilin1 (*13*). In contrast, the *NL-G-F* is a KI model with normal levels of APP. Thus, the differences between the findings presented here and the previous study could be explained by the models analyzed.

Understanding how risk factors contribute to the pathogenesis of AD is critical for identifying therapeutic targets to prevent or ameliorate synaptic dysfunction and synapse loss in this condition. Our findings uncover the impact of a genetic variant of *LRP6* associated with LOAD on synapse vulnerability with age and in the context of AD. Thus, these findings further strengthen the link between deficient Wnt signaling and synapse loss in the aging and AD brain.

## MATERIALS AND METHODS

### Animals

Experiments with mice were carried out under personal and project licenses granted by the U.K. Home Office in accordance with the Animals (Scientific Procedures) Act 1986 and were approved by the University College London ethical committee. Animals were housed in ventilated cages with a 12-hour light/12-hour dark cycle and ad libitum access to food and water. Both male and female animals were used. Ages are specified in the figure legends.

### Generation of *Lrp6-val* mutant mice

Single-strand oligonucleotides (ssODNs) were synthesized by Integrated DNA Technologies (IDT) (ssODN, CATCAGAGGCAGTCTCAGGCTGTGGCTTTGGAACATACCCTTTCTCGGGGTTTACCACAACGGCTCAGGTCTGTCTTGCTCGCCTTTTAGAACCACTCCAACTGATCGTCCATCTAATC). ssODNs were positioned adjacent to the guide RNA (gRNA) site: ACAGACCTCGAGCCATTGTGG. gRNA oligonucleotides were synthesized, and the two strands were annealed and cloned using Bsa I into a vector containing the gRNA backbone and a T7 promoter for RNA production. For Cas9 mRNA production, the vector from (*55*) was modified to contain the T7 promoter. Four- to 5-week-old C57BL/6NTac females were superovulated by injection of 5 IU of pregnant mare’s serum, and 48 hours later, 5 IU of human chorionic gonadotrophin (HCG) was injected. Females were mated with C57BL/6NTac males. Cumulus oocyte complexes were dissected from oviducts 21 to 22 hours after HCG and treated with hyaluronidase. Fertilized one-cell embryos were maintained at 37°C in KSOM media before cytoplasmic injection. Twenty-four to 27 hours after HCG, Cas9 mRNA (50 ng/μl), gRNA (25 ng/μl) (each), and oligonucleotide (100 ng/μl) were injected into the cytoplasm of fertilized one-cell embryos held in FHM medium. Viable embryos were transferred on the same day by oviducal embryo transfer into 0.5-day postcoital pseudo-pregnant female F1 (CBA/C57BL/6J) recipients. Homozygous *Lrp6-Val* C57BL/6NTac mice were backcrossed to C57BL/6J mice.

### Genotyping

*Lrp6-val* mice were crossed to the *Thy1-GFP* mice or *NL-G-F* model to obtain *Lrp6-val;Thy1-GFP* mice and *NL-G-F;Lrp6-val* mice, respectively. Genotyping was performed on ear biopsies using the following primers: *Lrp6* WT (forward: GATACGTTGCTTTAATGCCTTTAGCAAGACAGACCTCGAGCAA), *Lrp6-val* (forward: TGGCGGCAAGACAGACCTCGAGCAG), *Lrp6* (WT and Val) (reverse: AACGCGCAACGAAGGGTGAGGAGGCATCA), *NL-G-F* (5′-ATCTCGGAAGTGAAGATG-3′, 5′-ATCTCGGAAGTGAATCTA-3′, 5′-TGTAGATGAGAACTTAAC-3′, and 5′-CGTATAATGTATGCTATACGAAG-3′) (*49*), and GFP (forward: 5′-TCTGAGTGGCAAAGGACCTTAGG-3′; reverse: 5′-CGCTGAACTTGTGGCCGTTTACG-3′) (*37*).

### Hippocampal culture and transfection

Rat hippocampal cultures were prepared from embryonic day 18 (E18) embryos from Sprague-Dawley rats as previously described (*12*, *19*). Cultures were maintained for 13 to 14 days in vitro (DIV) to assess presynaptic terminals or until 21 DIV to investigate dendritic spines or to analyze the impact of sFRP1 on presynaptic terminals.

Mouse hippocampal neurons were prepared from E15.5 to E16.6 WT or *Lrp6-val* mice and maintained until 12 to 21 DIV. Neurons were treated with recombinant Wnt7a (100 or 200 ng/ml; PeproTech, 120-31), recombinant Wnt3a (200 ng/ml; R&D Systems), or with bovine serum albumin (BSA; control vehicle) for 3 hours on DIV 12 for analyses of synapses.

Rat hippocampal neurons were transfected using either Amaxa nucleofection before plating or with calcium phosphate transfection at 7 to 9 DIV with DNA constructs expressing enhanced green fluorescent protein (EGFP)–actin, human *LRP6* WT or human *LRP6-Val*, and MESD (mesoderm development LRP chaperone protein), which is required for maturation and trafficking of LRP6 (*56*). Control neurons were transfected with EGFP-actin only. Neurons (DIV 20) were treated with recombinant sFRP1 (1 μg/ml; R&D Systems) overnight for analyses of presynaptic terminals.

### HeLa cell culture and transfection

HeLa cells were grown in Dulbecco’s modified Eagle’s medium (DMEM; Gibco) supplemented with 10% fetal bovine serum and 1% penicillin/streptomycin (Gibco) and maintained at 37°C and 5% CO_2_. Cells were seeded on 12-mm glass coverslips at a density of 13.4 × 10^3^ cells/cm^2^ for PLA assays, 13.95 × 10^3^ cells/cm^2^ for analyses of pLRP6, and at 18.6 × 10^3^ cells/cm^2^ for surface biotinylation.

Cells were transfected with plasmids encoding EGFP, Fz5-HA, MESD, and *LRP6* WT or *LRP6-Val* using Lipofectamine 3000 (Invitrogen) and Opti-MEM for 4 hours according to the manufacturer’s protocol. The transfection medium was then replaced with Opti-MEM. Forty-eight hours after transfection, cells were treated with serum-free DMEM containing recombinant Wnt7a (200 ng/ml; PreproTech, 120-31) or BSA (control) for 30 or 60 min.

### List of plasmids

*LRP6* WT (Addgene, plasmid no. 27242) and Fz5-HA were gifts from X. He. MESD-Flag was a gift from B. Holdener. EGFP-actin was a gift from Y. Goda. *LRP6-Val-HA* was a gift from R. T. Moon. The untagged *LRP6-Val* plasmid used in this paper was generated using constructs provided by R. T. Moon.

### Brain section preparation

For cryostat sections used for immunostaining, 4% paraformaldehyde (PFA) fixed brains were immersed in 30% sucrose and frozen in precooled 2-methylbutane. Thirty- to 50-μm sagittal sections were cut using a cryostat and stored at −20°C. Vibratome sections were prepared as previously described (*57*). Briefly, brains were rapidly dissected and immersed in artificial cerebrospinal fluid (ACSF), and 250- to 300-μm-thick sagittal brain slices were cut. Then, slices were fixed in 4% PFA/4% sucrose in phosphate-buffered saline (PBS).

### Immunofluorescence staining

Immunofluorescence staining was performed as previously described (*57*). Slices were permeabilized and blocked in 0.5% Triton X-100 + 10% donkey serum in PBS for 3 to 4 hours at room temperature (RT) and then incubated with primary antibodies overnight at 4°C. Secondary antibodies (1:500; Alexa Fluor, Invitrogen) were incubated for 2 hours at RT. Slices were incubated in DAPI, washed with PBS, and mounted with Fluoromount-G (SouthernBiotech).

Cultured hippocampal neurons were fixed in 4% PFA with 4% sucrose in PBS for 20 min at RT. Neurons were permeabilized in 0.05% Triton X-100 in PBS for 5 min, blocked in 5% BSA for 1 hour, both at RT, and then incubated with primary antibodies overnight at 4°C. Secondary antibodies (1:600; Alexa Fluor, Invitrogen) were incubated for 1 hour at RT. Neurons were incubated with DAPI, washed with PBS, and mounted with FluorSave (Millipore).

### Proximity ligation assay

PLA was performed according to the manufacturer’s protocol (Sigma-Aldrich). Briefly, cells were washed with PBS, fixed with warm 4% PFA for 15 min, and then blocked for 60 min at 37°C with the Duolink blocking solution. Cells were incubated overnight at 4°C with anti-LRP6 (R&D Systems, AF1505) and anti-HA (Sigma-Aldrich, H6908) primary antibodies in Duolink antibody diluent solution (1:800). Cells were washed three times with the Duolink wash buffer A and incubated with the anti-rabbit MINUS and anti-goat PLUS PLA probes in Duolink antibody diluent solution for 1 hour at 37°C. After two washes in wash buffer A, cells were incubated with the Duolink ligation solution for 30 min at 37°C. After another two wash buffer A washes, cells were incubated with the Duolink amplification solution for 1 hour 40 min at 37°C. Cells were washed 2 × 10 min in wash buffer B and for 1 min in 0.01× wash buffer B. Cells were then permeabilized for 10 min with 0.1% Triton X-100/PBS and blocked in 5% BSA for 1 hour. Anti-GFP (1:500; Millipore, 06-896) primary antibody was added for 1 hour at RT, followed by three PBS washes and the addition of Alexa Fluor 488 chicken for 1 hour at RT. After 3× PBS washes, coverslips were mounted using 5 μl per coverslip of Duolink in situ mounting medium with DAPI.

### List of primary antibodies

The following primary antibodies were used: APP (6E10) (Novus Biotech, NBP2-62566, RRID:AB_2917960), Aβ (BioLegend, 803001, RRID:AB_2564653), β-actin (Cell Signaling Technology, 4970, RRID:AB_2223172), Bassoon (Novus Biologicals, NB120-13249), Bassoon (Synaptic Systems, 141016, RRID:AB_2661779), GFP (Millipore, 06-896, RRID:AB_310288), GFP (Invitrogen, A-6455), HA (Sigma-Aldrich, H6908, RRID:AB_260070), HA (Roche, 11867423001, RRID:AB_390918), Homer1, (Synaptic Systems, 160002, RRID:AB_2120990), Homer1, (Synaptic Systems, 160003, RRID:AB_887730), Homer1, (Synaptic Systems, 160006, RRID:AB_263122), LRP6 (Abcam, ab134146, RRID:AB_2895164), LRP6 (R&D Systems, AF1505, RRID:AB_2266025), LRP6 (Cell Signaling Technology, 2560, RRID:AB_2139329), LRP6 (Cell Signaling Technology, 3395, RRID:AB_1950408), pLRP6 (Cell Signaling Technology, 2568, RRID:AB_2139327), MAP2 (Abcam, ab5392, RRID:AB_2138153), MAP2 (Abcam, ab92434, RRID:AB_2138147), NeuN, (Cell Signaling Technology, 12943, RRID:AB_2630395), PSD-95 (Millipore, MAB1598, RRID:AB_94278), α-tubulin (Sigma-Aldrich, T9026, RRID:AB_477593), vGlut1 (Millipore, AB5905, RRID:AB_2301751), and vinculin (Sigma-Aldrich, V4505, RRID:AB_477617).

### Confocal microscopy

Images were acquired on a Leica SP8 or an Olympus FV1000 inverted confocal microscope. For analyses of synaptic puncta and dendritic spines in brain sections, three images (stacks) were acquired per brain section, and three brain sections were analyzed per animal. For hippocampal cultures, 6 to 13 images (stacks) were acquired per condition. Each stack comprised 8 to 11 equidistant planes, 0.2 to 0.25 μm apart, and were acquired using a 63× 1.40–numerical aperture (NA) oil objective. For analyses of NeuN staining, a stack of 31 equidistant planes, 0.5 μm apart, was acquired for each brain section with a 10× 0.40-NA objective on a Leica SP8. For plaque analyses of 2-month-old mice, images were acquired on a Leica SP5. For each brain section, one stack of 25 equidistant planes, 0.99 μm apart, was acquired using a 10× 0.30-NA objective. For analyses of plaques in 7- and 10-month-old mice, a tile scan per brain section was acquired with a 2× 0.75-NA objective on a Leica SP8. Each stack comprised 59 equidistant planes for 7-month-old mice and 144 equidistant planes for 10-month-old mice, 0.35 μm apart. For the PLA experiments and analyses of pLRP6 in HeLa cells, stacks of 9 to 10 equidistant planes with a *z* step of 0.5 μm were acquired using a 40× oil objective on a Leica SP8. Two to three coverslips per experimental condition were imaged with two to five images per coverslip acquired.

### Structured illumination microscopy

SIM was performed on a Zeiss Elyra S.1 microscope with a 63× oil-immersion objective (NA 1.40). Z-stacks of 15 to 20 equidistant planes were acquired. For each field of view, nine images were acquired using three different rotations and phases of structured illumination (a grid pattern) on the sample.

### Electron microscopy

Sagittal brain sections of 200-μm thickness were cut on a vibratome and then fixed in 2% PFA/2.5% glutaraldehyde solution overnight at 4°C. Samples were postfixed in 1% OsO_4_ for 1 hour at 4°C and stained with 1% thiocarbohydrazide for 20 min at RT, 2% OsO_4_ for 30 min at RT, 1% uranyl acetate overnight at 4°C, and lead aspartate for 30 min at 60°C. Next, slices were dehydrated in graded alcohol and embedded in resin. Ultrathin sections (70 nm) were then cut using a diamond ultra 45° knife (Diatome) on a Leica UC7 ultramicrotome and collected on 2 mm–by–1 mm slot grids. Images were acquired on a transmission electron microscope (T12 Tecnai Spirit Bio-Twin, FEI), each covering 5.8 μm^2^ at 0.77 nm/pixel.

### Image analyses

Image analyses were performed using Volocity software (PerkinElmer). For hippocampal cultures and brain sections, customized thresholding protocols were used to detect pre- and postsynaptic puncta. Synapses were quantified as colocalized pre- and postsynaptic puncta. Analyses of dendrite spines were performed blind to the genotype or treatment. Dendritic spines were measured manually along three to four sections of dendrite. Spine size was quantified using the line tool by measuring the maximum spine head width. For the PLA experiment, only transfected cells were selected (on the basis of GFP signal), and the total PLA signal intensity was quantified and divided by the number of cells analyzed. For analyses of pLRP6 levels, cells expressing GFP and *LRP6* were analyzed. Total pLRP6 intensity was normalized to total LRP6 intensity in each cell. For SIM images, synapses were identified manually, and the colocalization of LRP6 with synaptic markers was determined using Volocity. For neuronal number, the number of NeuN and DAPI-positive cells was quantified using Volocity and divided by the total number of DAPI-positive cells. In *NL-G-F* and *NL-G-F;Lrp6-val* mice, plaques were manually counted at 2 months, blind to the genotype. At 7 months, plaque analyses were performed, blind to the genotype, using Fiji as previously described (*58*). Images were thresholded, and then, the particle analysis tool was used to count the number of plaques and the percent coverage area of Aβ. For EM, high-magnification images were used to manually count synaptic vesicles within 200 nm of the active zone. PSD length was quantified using a line tool in Fiji. Vesicle number was normalized to PSD length.

### Electrophysiological recordings

Electrophysiological recordings were performed in 7- to 8-month-old and in 12- to 13-month-old male and female mice. Acute transverse hippocampal slices (300 μm thick) from WT control mice and homozygous *Lrp6-val* mice were cut with a Leica VT-1000 vibratome in ice-cold ACSF bubbled with 95% O_2_/5% CO_2_ containing NaCl (125 mM), KCl (2.4 mM), NaHCO_3_ (26 mM), NaH_2_PO_4_ (1.4 mM), d-(+)-glucose (20 mM), CaCl_2_ (0.5 mM), and MgCl_2_ (3 mM). Brain slices from 12- to 13-month-old mice were then transferred for 5 min into a series of three different oxygenated (95% O_2_/5% CO_2_) chambers in the same ACSF base but with gradual temperature and component variations: (i) 21°C, MgCl_2_ (1 mM) and CaCl_2_ (0.5 mM) and then placed at 36°C for 5 min; (ii) 36°C, MgCl_2_ (1 mM) and CaCl_2_ (1 mM); and (iii) 36°C with MgCl_2_ (1 mM) and CaCl_2_ (2 mM) before cooling to 21°C for at least 1 hour before recordings. Brain slices were placed in a chamber on an upright microscope and constantly perfused with NaCl (125 mM), KCl (2.4 mM), NaHCO_3_ (26 mM), NaH_2_PO_4_ (1.4 mM), d-(+)-glucose (20 mM), MgCl_2_ (1 mM), and CaCl_2_ (2 mM) supplemented with 10 μM bicuculline to block γ-aminobutyric acid currents at RT.

Whole-cell patch-clamp recordings were made from pyramidal cells in the CA1 region voltage-clamped at −60 mV using patch pipettes with a resistance of 4 to 8 megohm when filled with a cesium gluconate pipette solution composed of d-gluconic acid lactone (130 mM), Hepes (10 mM), EGTA (10 mM), NaCl (10 mM), CaCl_2_ (0.5 mM), MgCl_2_ (1 mM), adenosine 5′-triphosphate (1 mM), and guanosine 5′-triphosphate (0.5 mM), and QX314 (5 mM) adjusted to pH 7.2 with CsOH. To evoke postsynaptic currents, a bipolar concentric stimulating electrode (FHC) connected to a Grass S48 stimulator was placed around 100 to 200 μm from the patched cell. Cell I/O recordings were made with the stimulus pulse varied between 9 and 50 V with a pulse width of 0.1 ms and delivered at a rate of 0.1 Hz. At least three responses per stimulus intensity were averaged per cell. PPR stimuli were delivered at a rate of 0.2 Hz with varying ISIs, ranging from 50 to 200 ms. The stimulus intensity was adjusted for each cell to elicit ∼50% of the maximal response. PPR was calculated as the ratio of the peak amplitude of the second response over the first response, and at least seven responses were averaged per cell for each ISI. For RRP size, initial fusion efficiency, and SV recycling rate calculation, CA1 cell EPSCs were recorded in response to 3-s duration trains of stimulation at 20 Hz and estimated as previously described (*40*, *43*).

The following two equations were used to estimate RRP size, fusion efficiency (*fe*), and vesicle recycling rate (α) using cumulative charge (*43*) in analysis of 20-Hz stimulus-evoked trains of EPSCs

fe=r(1)r(∞)(1−exp.(−α⁡Δt))fe=r(1)r(∞)(1−exp.(−α⁡Δt)) (1)

fe=r(1)∑si=1r(i)exp.(−α⁡(S−i)Δt)fe=r(1)∑i=1sr(i)exp.(−α⁡(S−i)Δt) (2)

where *r*(1) is the charge of the first EPSC in the train, *r*(*i*) is the charge passed by the *i*th EPSC, *r*(∞) was calculated from the average charge of the last 10 EPSCs in the train, and Δ*t* is the stimulus interval in the train. The RRP was estimated as RRP = *r* (1)/*fe*.

Currents were recorded using an Axopatch 200B amplifier and low-pass–filtered at 1 kHz and digitized at 10 kHz. Online monitoring of the data was performed using WinEDR and offline analysis using both WinEDR and WinWCP software (freely available online at http://spider.science.strath.ac.uk/sipbs/software_ses.htm).

### Surface biotinylation and Western blots

Surface biotinylation was performed using Sulfo-NHS-LC-LC-Biotin (Thermo Fisher Scientific, EZ-Link Sulfo-NHS-LC-LC-Biotin) and streptavidin agarose beads (Thermo Fisher Scientific). Samples were run on an 8% SDS–polyacrylamide gel electrophoresis (SDS-PAGE) gel. Hippocampal homogenates from WT and *Lrp6-val* mice at 4 months old were resolved on 8 to 12% SDS-PAGE gels. Two bands were observed for Fz5-HA. Both bands were quantified as these bands are likely to represent changes in the glycosylation of this receptor at the surface (*59*).

### Enzyme-linked immunosorbent assay

*NL-G-F* and *NL-G-F;Lrp6-val* hippocampal tissue was homogenized in radioimmunoprecipitation assay (RIPA) buffer, followed by guanidine hydrochloride. Aβ42 peptides were quantified using a human Aβ 1-42 ELISA kit (Wako) following the manufacturer’s instructions.

### Quantitative polymerase chain reaction

RNA was extracted from frozen hippocampal tissue using TRIzol (Life Technologies) and the Direct-zol RNA MiniPrep Kit (Zymo Research), following the manufacturer’s instructions. First-strand complementary DNA (cDNA) synthesis was performed with the RevertAid H Minus First Strand cDNA Synthesis Kit (Thermo Fisher Scientific), following the manufacturer’s instructions. qPCR was performed using GoTaq qPCR Master Mix (Promega). *Gapdh*, *Canx*, or *Rpl13a* was used as housekeeping genes. The following primers were used: *Lrp6* (forward: 5′-TCTTGTGGTTGTCTGGTGTGGAG-3′; reverse: 5′-AGAAGACATATCAGAAAATGCAGGAGG-3′ or forward: 5′-AAGCTGCTGGAGAATGGAAA-3′; reverse: 5′-CCAAAGAAATTCGCCTCAAG-3′), *Axin2* (forward: 5′-GAGGGACAGGAACCACTCG-3′; reverse: 5′-TGCCAGTTTCTTTGGCTCTT-3′), *Fz5* (forward: 5′-ACATGGAACGATTCCGCTAC-3′; reverse: 5′-TCCCAGTGACACACACAGGT-3′), *Wnt7a* (forward: 5′-TTTCTCAGCCTGGGCATAGT-3′; reverse: 5′-CCAGAGCTACCACCGAAGAG-3′), *Wnt7b* (forward: 5′-GCCTTCACCTATGCCATCAC-3′; reverse: 5′-CCTTCCGCCTGGTTGTAGTA-3′), *Gapdh* (forward: 5′-CGTCCCGTAGACAAAATGGT-3′; reverse: 5′-TCAATGAAGGGGTCGTTGAT-3′ or forward: 5′-AGACAGCCGCATCTTCTTGT-3′; reverse: 5′-CTTGCCGTGGGTAGAGTCAT-3′), *Canx* (forward: 5′-CCCACATAGGAGGTCTGACA-3′; reverse: 5′-GCTAGGAATGGAGGAGATCCA-3′), and *Rpl13a* (forward: 5′-GACTCCTGGTGTGAACCCA-3′; reverse: 5′-CTCTACCCACAGGAGCAGT-3′).

### Statistical analyses

All results are presented as means ± SEM. Statistical analyses were performed in GraphPad Prism (version 9) or SPSS (IBM, version 27). Normality was assessed with Shapiro-Wilk or Kolmogorov-Smirnov tests. Normally distributed data were analyzed using *t* tests for two conditions, one-way analysis of variance (ANOVA) for two or more conditions, or two-way ANOVA for experiments with two independent variables. Post hoc tests are detailed in the figure legends. Nonnormally distributed data were analyzed with nonparametric tests such as Kruskal-Wallis or Mann-Whitney. Statistical significance was accepted as *P < 0.05, **P < 0.01, and ***P < 0.001.

## Notes

View/request a protocol for this paper from *Bio-protocol*.
