# Supplementary material for: A genetic variant of the Wnt receptor LRP6 accelerates synapse degeneration during aging and in Alzheimer’s disease
Source: Sci Adv. 2023 Jan 13;9(2):eabo7421. doi: 10.1126/sciadv.abo7421 (PMC10624429; doi:10.1126/sciadv.abo7421)
Supplement: Supplementary file 1 — Figs. S1 to S12 [file sciadv.abo7421_sm.pdf]

Science Advances
AAAS

Supplementary Materials for

A genetic variant of the Wnt receptor LRP6 accelerates synapse degeneration during aging and in Alzheimer's disease

Megan E. Jones *et al.*

Corresponding author: Patricia C. Salinas, p.salinas@ucl.ac.uk

Sci. Adv. **9**, eabo7421 (2023)
DOI: 10.1126/sciadv.abo7421

This PDF file includes:

Figs. S1 to S12

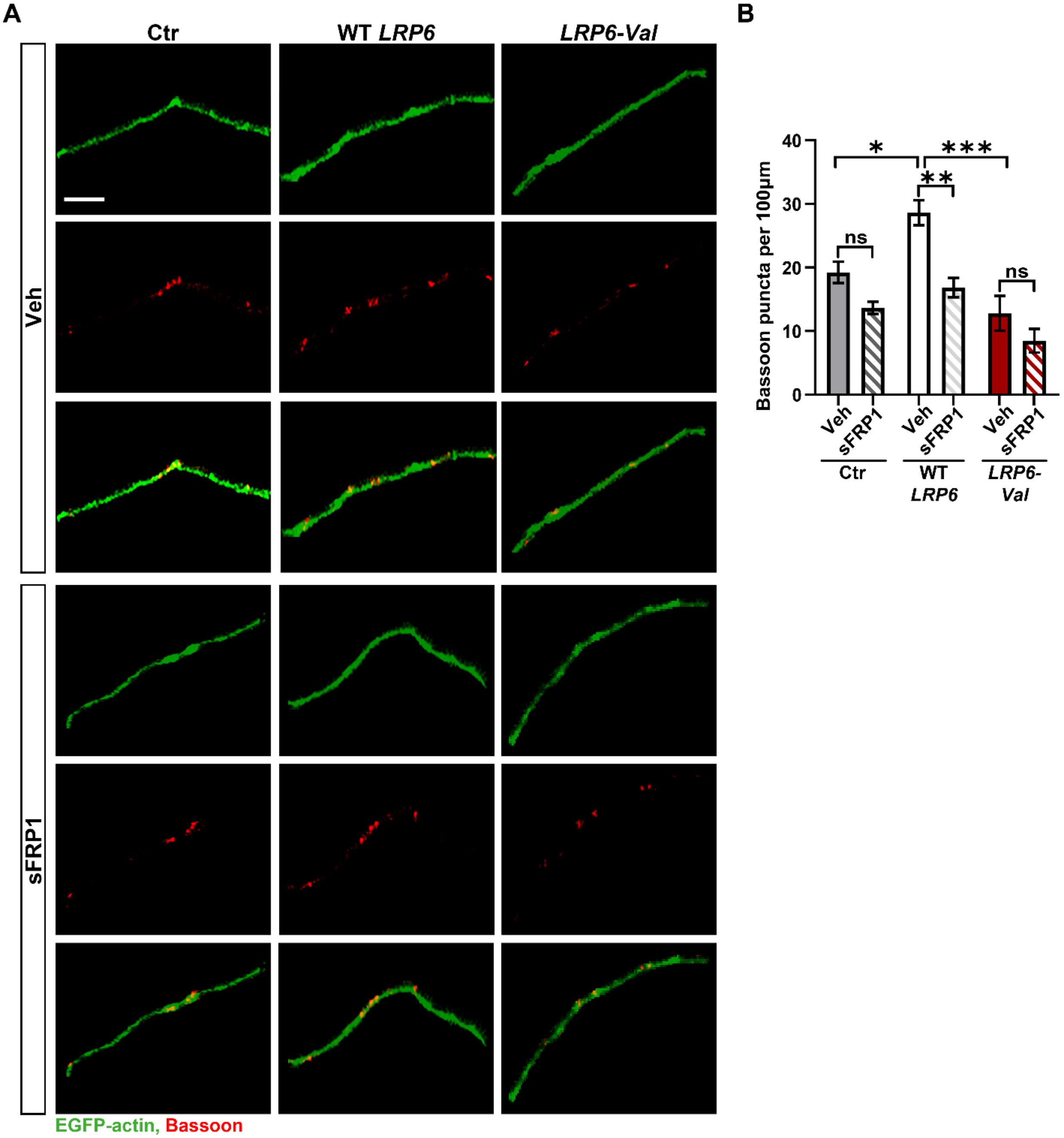

A
Ctr
WT LRP6
LRP6-Val
Veh
sFRP1
EGFP-actin, Bassoon
B
Bassoon puncta per 100µm
40
30
20
10
0
*
***
**
ns
ns
Veh
sFRP1
Veh
sFRP1
Veh
sFRP1
Ctr
WT LRP6
LRP6-Val

Figure S1

Figure S1. Inhibition of endogenous Wnts with sFRP1 blocks the effect of WT *LRP6* expression on presynaptic assembly. A) Confocal images of bassoon puncta (red) on isolated axons of neurons expressing EGFP-actin (green) alone, or with EGFP-actin and human WT *LRP6* or *LRP6-Val* exposed to recombinant sFRP1 or a control vehicle (veh). Scale bar = 5 μm. **B**). sFRP1 blocked the increase in the number of bassoon puncta in cells expressing WT *LRP6.* sFRP1 had no effect on cells expressing *LRP6-Val*. N = 3 independent cultures, 6-9 axons imaged per culture. Two-way-ANOVA with Tukey's post-hoc test. * $p < 0.05$, ** $p < 0.01$, *** $p < 0.001$. Data are represented as mean ± SEM.

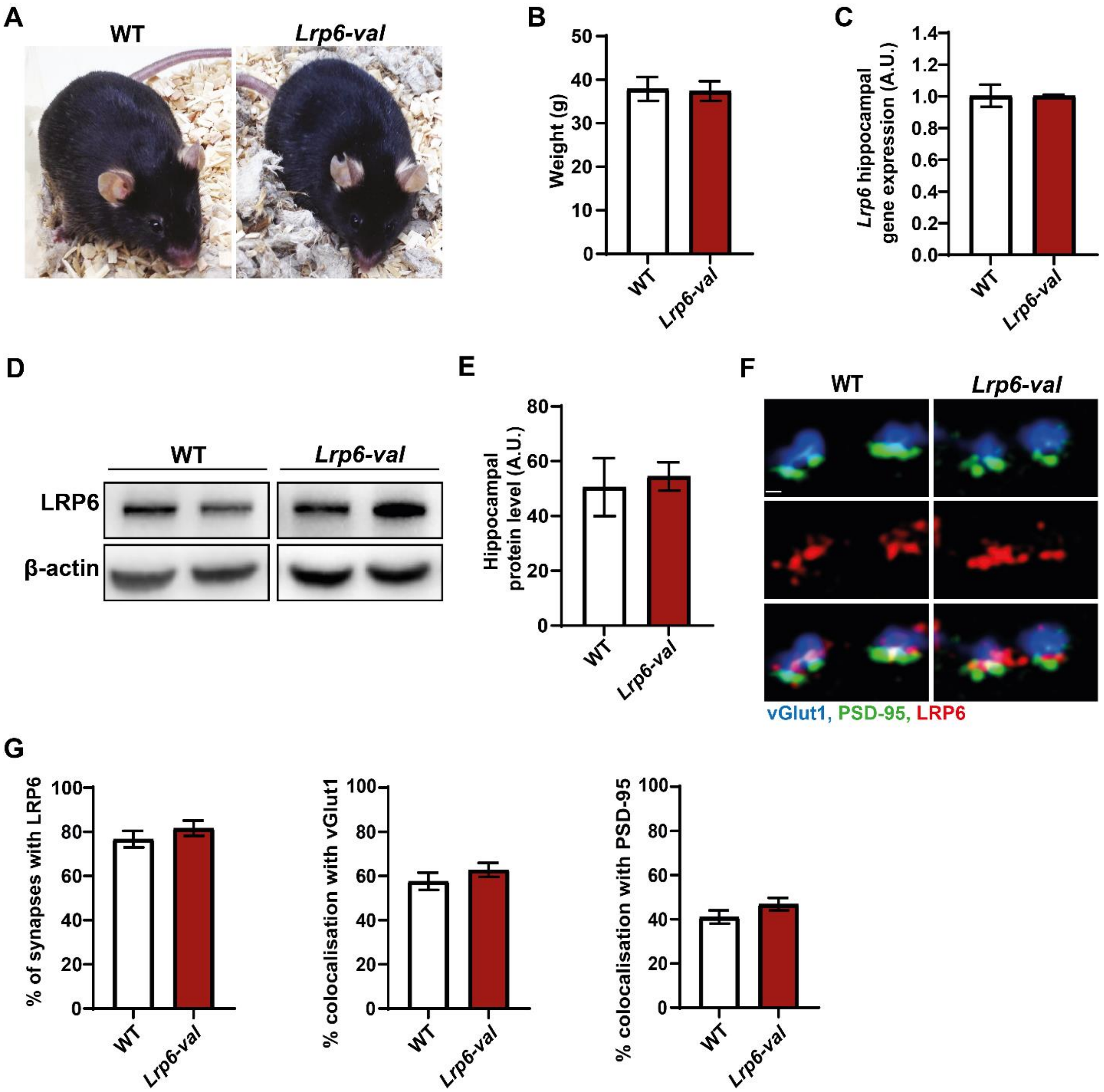
A
WT
Lrp6-val
B
Weight (g)
50
40
30
20
10
0
WT
Lrp6-val
C
Lrp6 hippocampal gene expression (A.U.)
1.4
1.2
1.0
0.8
0.6
0.4
0.2
0.0
WT
Lrp6-val
D
WT
Lrp6-val
LRP6
β-actin
E
Hippocampal protein level (A.U.)
80
60
40
20
0
WT
Lrp6-val
F
WT
Lrp6-val
vGlut1, PSD-95, LRP6
G
% of synapses with LRP6
100
80
60
40
20
0
WT
Lrp6-val
% colocalisation with vGlut1
100
80
60
40
20
0
WT
Lrp6-val
% colocalisation with PSD-95
100
80
60
40
20
0
WT
Lrp6-val

Figure S2

Figure S2. Characterisation of homozygous *Lrp6-val* knock-in mice. **A**) Images of WT and *Lrp6-val* mice at 7 months showed that these mice developed normally and have no visible external abnormalities. **B**) Adult *Lrp6-val* mice had the same weights as control WT mice. Weights of male mice were measured at 4-8 months of age. WT N = 9. *Lrp6-val* N = 10. Unpaired T-test. **C**) Quantitative RT-PCR analyses of *Lrp6* mRNA levels in the hippocampus of WT and *Lrp6-val* mice at 3-4 months of age showed no changes in the expression of *Lrp6*. WT N = 5, *Lrp6-val* N = 6. Unpaired T-Test. **D**) Hippocampal LRP6 protein levels in WT and *Lrp6-val* mice at 4 months. **E**) No changes in LRP6 levels (normalised to β-actin) were observed between WT or *Lrp6-val* mice. N = 3 per genotype. Unpaired T-test. **F**) SIM images of excitatory synapses containing LRP6 (red) from WT and *Lrp6-val* hippocampal neurons showing vGlut1 (blue) and PSD-95 (green). Scale bar = 0.2 μm. **G**) LRP6 receptor exhibited similar pre- and post-synaptic localisation in WT and *Lrp6-val* mice. 2 independent cultures, 6-8 images per culture. Unpaired T-tests. Data are represented as mean ± SEM.

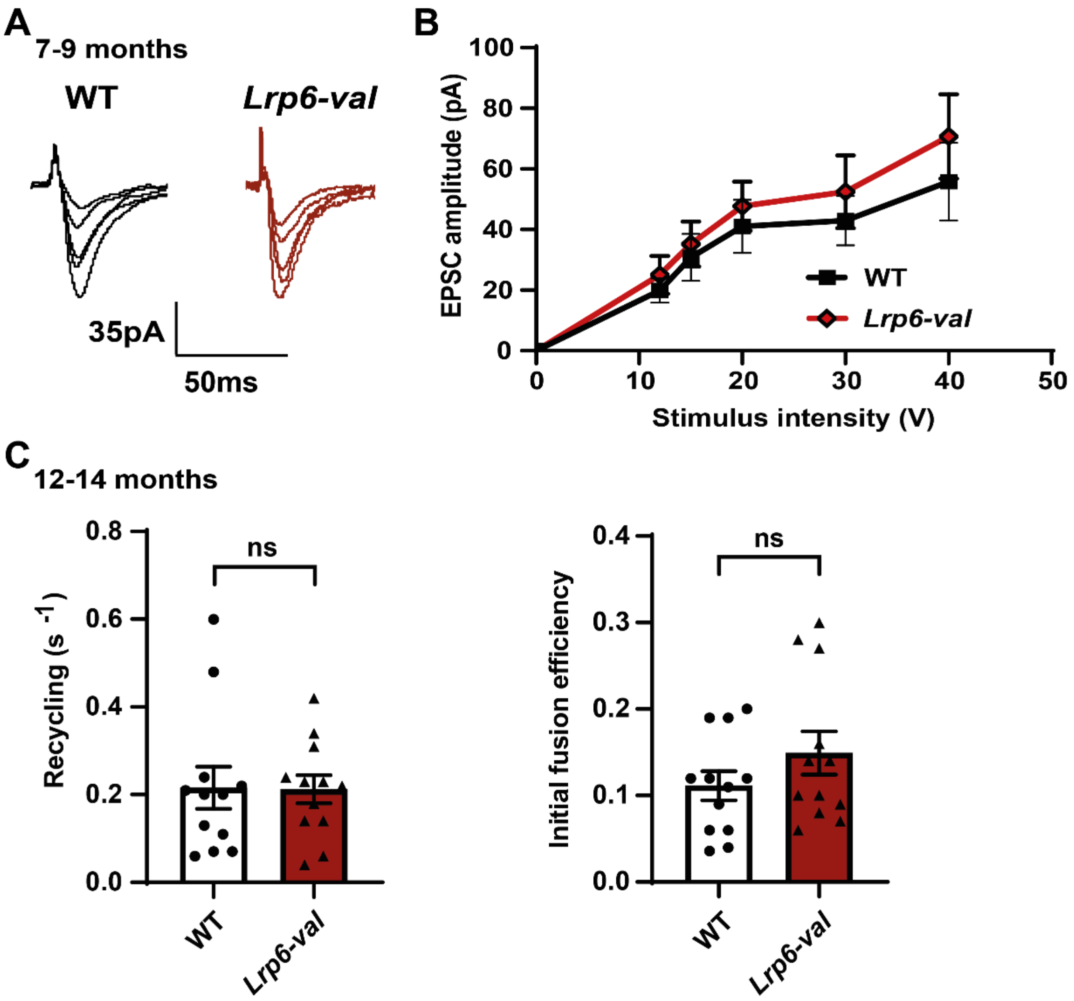

Figure S3

Figure S3. Basal synaptic transmission is unaffected in *Lrp6-val* mice at 7-9 months and vesicle recycling and initial fusion efficiency are unchanged in *Lrp6-val* mice at 12-14 months. **A**) Representative traces of post-synaptic currents elicited at different stimulation intensities. **B**) No differences were detected in the input-output curves at 7-9 months. N = 12-14 cells recorded from 4-5 animals per genotype. Repeated-measures one-way-ANOVA. **C**) Graphs display the recycling rate and initial fusion efficiency obtained from all cells. No differences were observed in *Lrp6-val* mice when compared to WT mice at 12-14 months. *N* = 12 cells from 4 animals per genotype. Unpaired Student's *T*-*t*est. Data are represented as mean ± SEM.

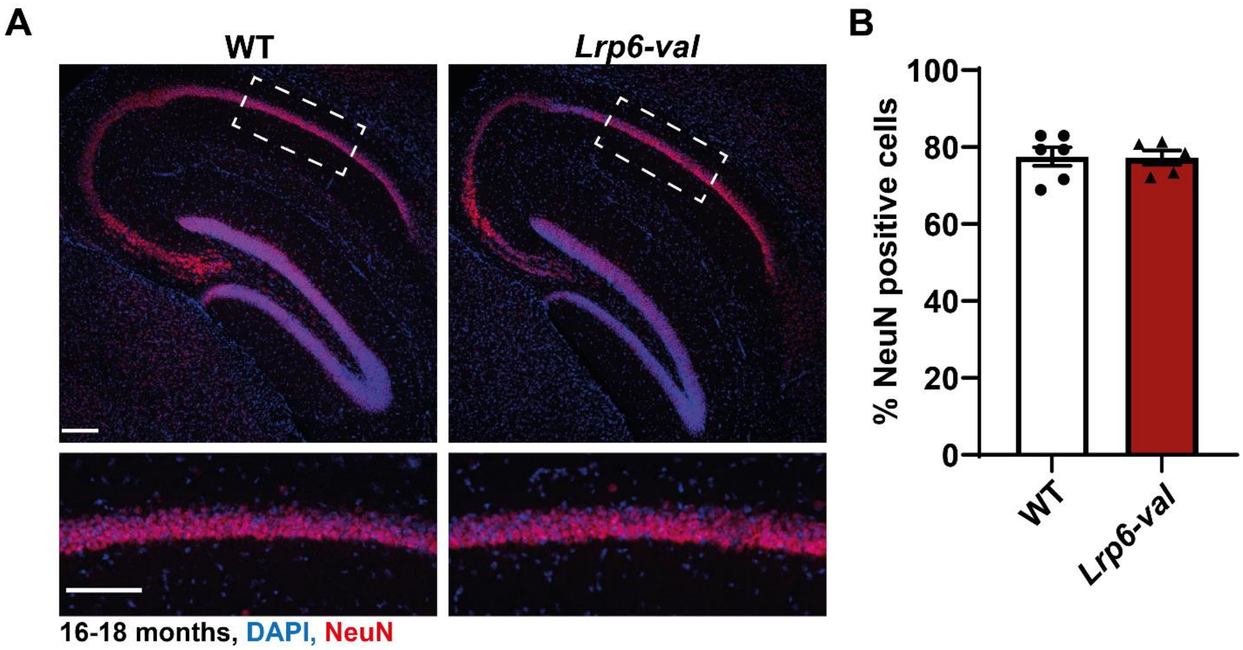

Figure S4

Figure S4. *Lrp6-val* mice do not display neuronal loss at 16-18 months. A) Confocal images of the hippocampus of WT and *Lrp6-val* mice labelled with DAPI (blue) and NeuN (red). Scale bar = 150 μm. Insets show higher magnification images of the CA1 region. Scale bar = 100 μm. **B**) Quantification revealed no differences in the percentage of NeuN positive cells between WT and *Lrp6-val* mice. WT N = 6, *Lrp6-val* N = 5. Unpaired T-test. Data are represented as mean ± SEM.

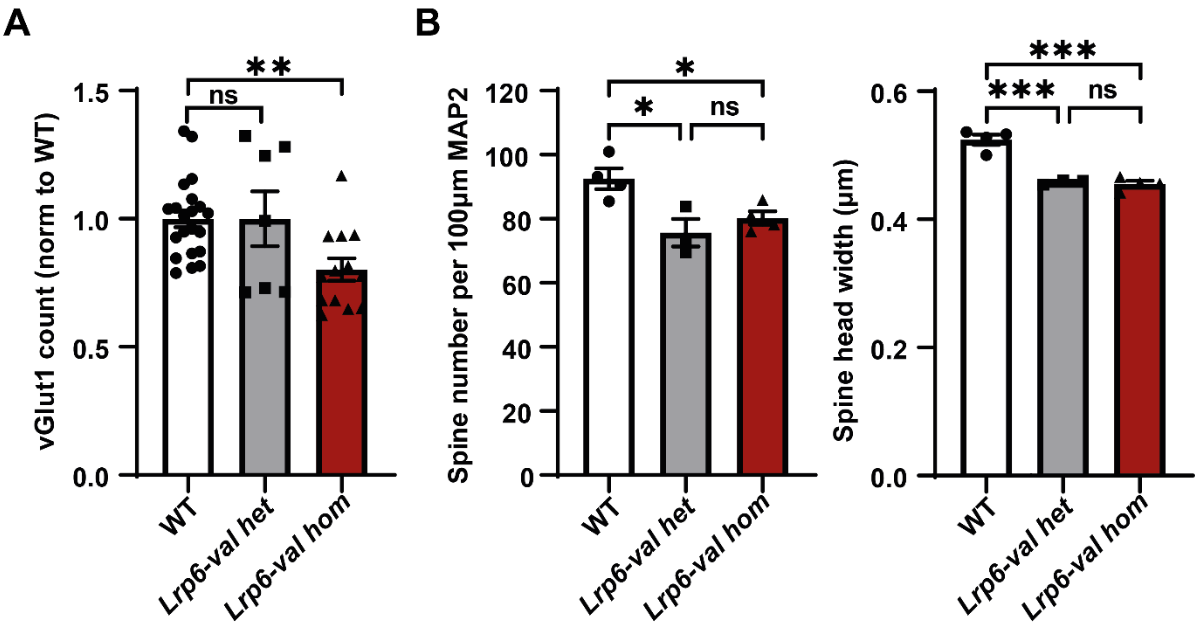

Figure S5

Figure S5. Heterozygous *Lrp6-val* mice display post-synaptic defects at 12-14 months. A) Quantification of vGlut1 puncta in the CA1 stratum radiatum of WT, *Lrp6-val* het and *Lrp6-val* hom mice at 12-14 months. *Lrp6-val* hom mice exhibited fewer vGlut1 puncta. WT N = 10, *Lrp6-val* het N = 4, *Lrp6-val* hom N = 10, 1-3 slices per brain. One-way-ANOVA with Tukey's post hoc test. **B**) Analyses of dendritic spines in *Lrp6-val* mice crossed to a *Thy1-GFP* reporter line at 12-14 months. Spine number and head width were decreased in both *Lrp6-val* het and *Lrp6-val* hom mice when compared to WT mice. WT N = 4, *Lrp6-val* het N = 3, *Lrp6-val* hom N = 4. One-way-ANOVA with Tukey's post hoc test. * $p < 0.05$, ** $p < 0.01$, *** $p < 0.001$. Data are represented as mean ± SEM.

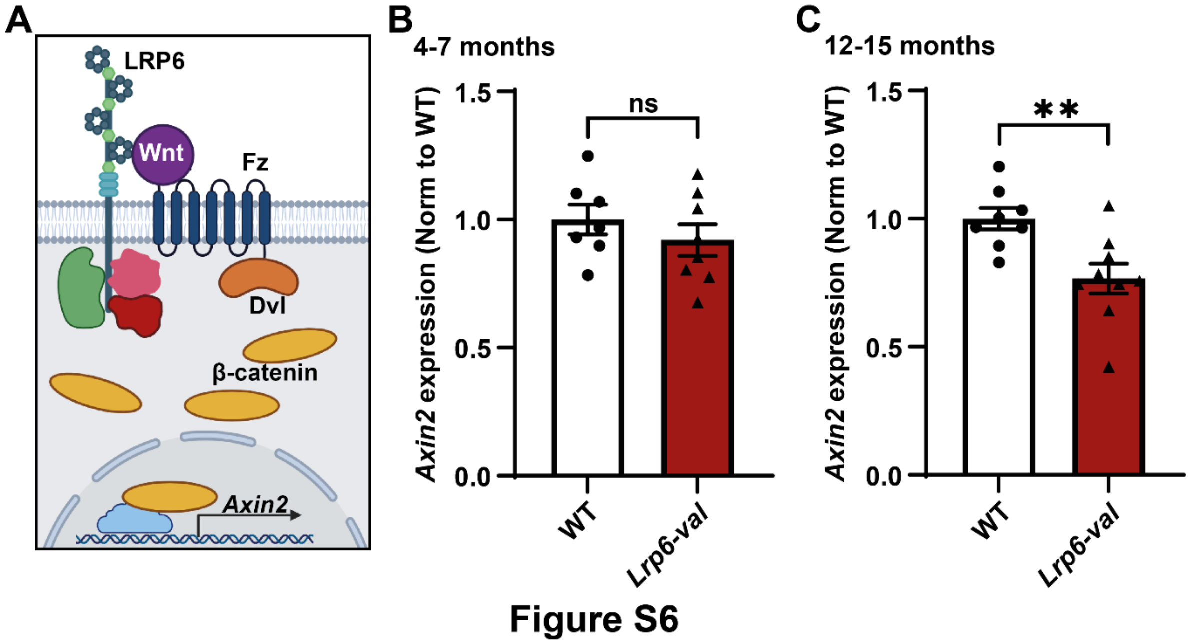

Figure S6. *Axin2* mRNA levels are reduced at 12-15 months in *Lrp6-val* mice. A) Schematic of the Wnt signalling pathway showing regulation of *Axin2* expression. **B** and **C**) Quantitative RT-PCR analyses of *Axin2* expression in the hippocampus of WT and *Lrp6-val* mice at 4-7 months (**B**) and 12-15 months (**C**). *Axin2* expression was reduced in *Lrp6-val* mice at 12-15 months but not before. 4-7 months: WT N = 7, *Lrp6-val* N = 8. 12-15 months: WT N = 8, *Lrp6-val* N = 9. Unpaired T-tests. ** $p < 0.01$. Data are represented as mean ± SEM.

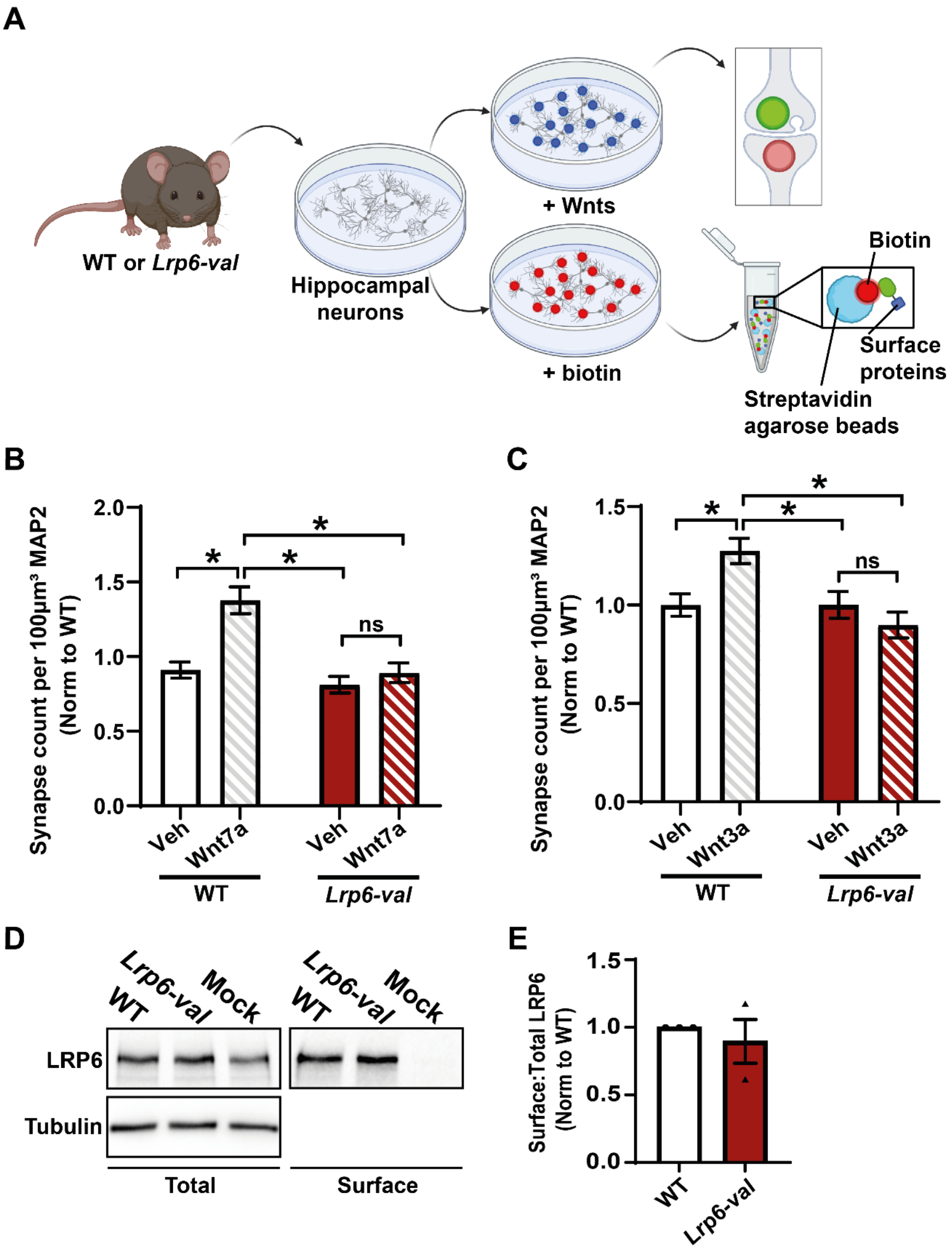

A
WT or Lrp6-val
Hippocampal neurons
+ Wnts
+ biotin
Biotin
Surface proteins
Streptavidin agarose beads
B
Synapse count per 100µm³ MAP2 (Norm to WT)
2.0
1.5
1.0
0.5
0.0
*
*
*
ns
Veh
Wnt7a
Veh
Wnt7a
WT
Lrp6-val
C
Synapse count per 100µm³ MAP2 (Norm to WT)
1.5
1.0
0.5
0.0
*
*
*
ns
Veh
Wnt3a
Veh
Wnt3a
WT
Lrp6-val
D
WT
Lrp6-val
Mock
WT
Lrp6-val
Mock
LRP6
Tubulin
Total
Surface
E
Surface:Total LRP6 (Norm to WT)
1.5
1.0
0.5
0.0
WT
Lrp6-val

Figure S7

Figure S7. *Lrp6-val* neurons do not respond to Wnt7a or Wnt3a to promote synapse formation. A) Diagram showing the isolation of primary neurons from WT and homozygous *Lrp6-val* mice to evaluate synapse number or surface levels of the receptor by surface biotinylation. Top: neurons were exposed to recombinant Wnt7a or Wnt3a for 3 hours prior to analyses of synapses by confocal microscopy. Bottom: neurons were incubated with biotin (red circles). Biotin bound surface proteins (Green and blue shapes) were pulled down with streptavidin-agarose beads (blue circles). **B**) Wnt7a (100ng/ml) increased the number of synapses in WT neurons, but Wnt7a had no effect on *Lrp6-val* neurons. N = 3 independent cultures, 8-11 images per culture. Two-way-ANOVA with Games-Howell post hoc test. * $p < 0.05$. **C**) Wnt3a increased synapse number in WT neurons but *Lrp6-val* neurons failed to respond to Wnt3a. N = 3 independent cultures, 8-10 images per culture. Two-way-ANOVA with Games-Howell post hoc test. * $p < 0.05$. **D**) Surface biotinylation analyses of LRP6 were performed on neurons isolated from WT and homozygous *Lrp6-val* mice. **E**) No differences in the ratio of surface to total LRP6 were observed. N = 3 independent cultures. Mann-Whitney. Data are represented as mean ± SEM.

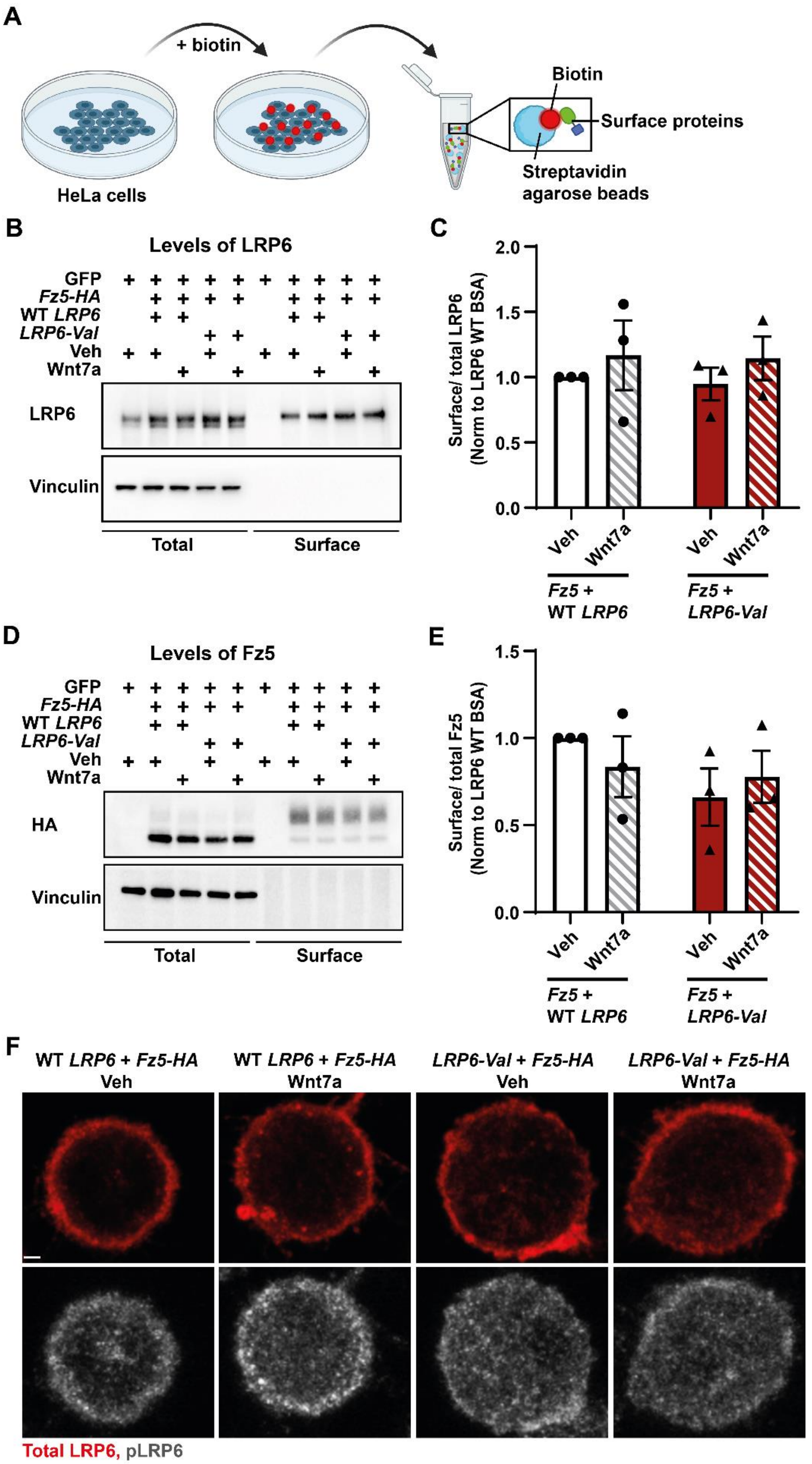

A
+ biotin
HeLa cells
Biotin
Surface proteins
Streptavidin
agarose beads
B
Levels of LRP6
GFP
Fz5-HA
WT LRP6
LRP6-Val
Veh
Wnt7a
LRP6
Vinculin
Total
Surface
C
Surface/ total LRP6
(Norm to LRP6 WT BSA)
2.0
1.5
1.0
0.5
0.0
Veh
Wnt7a
Veh
Wnt7a
Fz5 +
WT LRP6
Fz5 +
LRP6-Val
D
Levels of Fz5
GFP
Fz5-HA
WT LRP6
LRP6-Val
Veh
Wnt7a
HA
Vinculin
Total
Surface
E
Surface/ total Fz5
(Norm to LRP6 WT BSA)
1.5
1.0
0.5
0.0
Veh
Wnt7a
Veh
Wnt7a
Fz5 +
WT LRP6
Fz5 +
LRP6-Val
F
WT LRP6 + Fz5-HA
Veh
WT LRP6 + Fz5-HA
Wnt7a
LRP6-Val + Fz5-HA
Veh
LRP6-Val + Fz5-HA
Wnt7a
Total LRP6, pLRP6

Figure S8

Figure S8. The surface localisation of LRP6 is not affected in cells expressing *LRP6-Val*.

A) Schematic of surface biotinylation analyses of HeLa cells. **B**) Western blot analyses of LRP6 levels following surface biotinylation of cells expressing *Fz5-HA* and WT *LRP6* or *LRP6-Val* and treated with recombinant Wnt7a. **C**) No differences in the ratio of surface to total LRP6 were observed after Wnt7a treatment. N = 3 independent cultures. Two-way-ANOVA with Games-Howell post hoc test. **D**) Western blot analyses of Fz5-HA following surface biotinylation of HeLa cells expressing *Fz5-HA* and WT *LRP6* or *LRP6-Val* and treated with recombinant Wnt7a. We observed a higher molecular weight of Fz5-HA at the surface, which is probably due to changes in glycosylation of this receptor (*59*). **E**) The ratio of surface to total Fz5-HA were unchanged after Wnt7a treatment. Both bands observed for HA were quantified. N = 3 independent cultures. Two-way-ANOVA with Games-Howell post hoc test. **F**) Confocal images of HeLa cells expressing GFP, WT *LRP6* and *Fz5-HA* or GFP, *LRP6-Val* and *Fz5-HA*. Total LRP6 (red) and phosphorylated LRP6 (pLRP6) (grey). Scale bar = 2 μm. Data are represented as mean ± SEM.

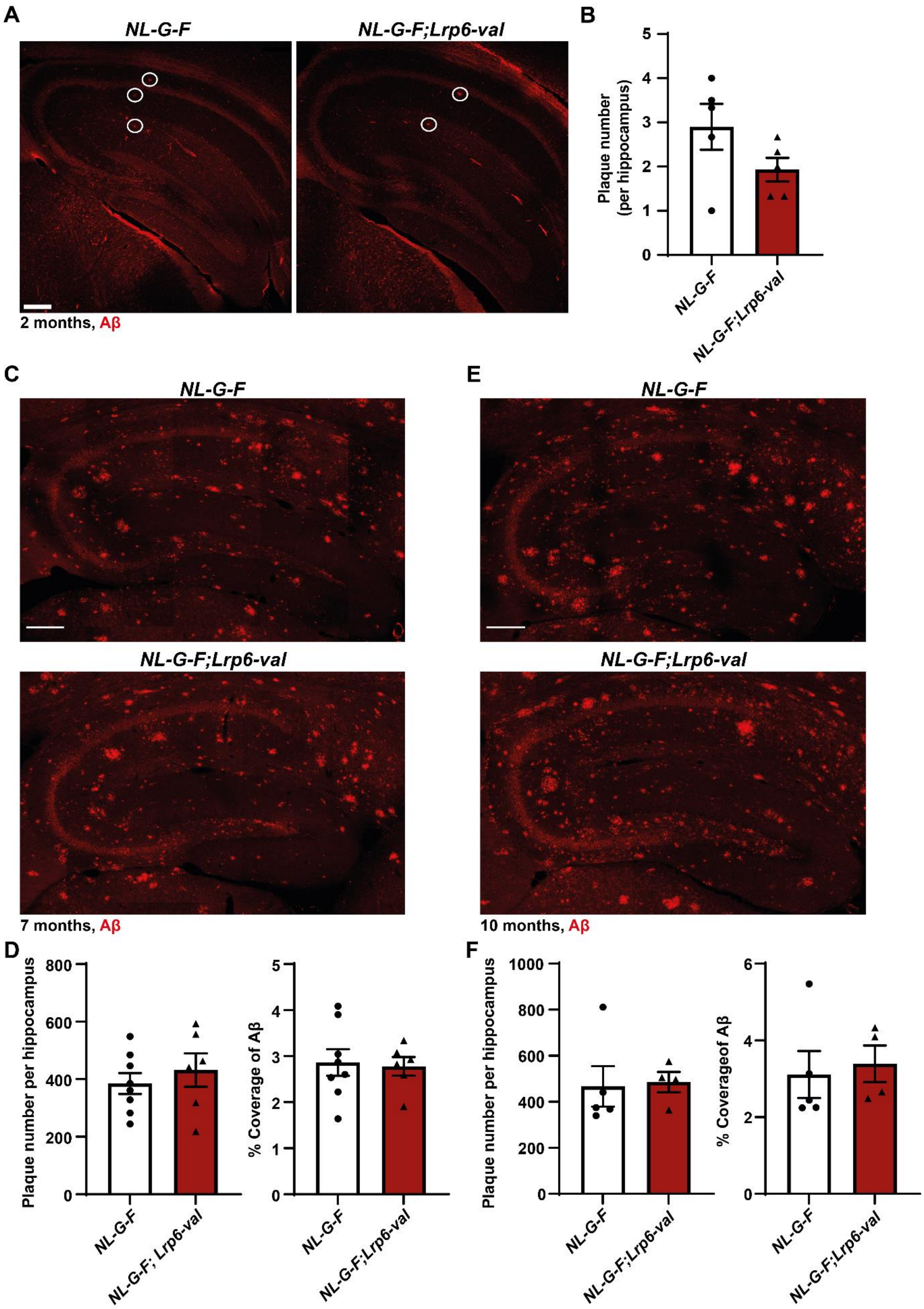
A
NL-G-F
NL-G-F;Lrp6-val
2 months, Aβ
B
Plaque number (per hippocampus)
0
1
2
3
4
5
NL-G-F
NL-G-F;Lrp6-val
C
NL-G-F
NL-G-F;Lrp6-val
7 months, Aβ
E
NL-G-F
NL-G-F;Lrp6-val
10 months, Aβ
D
Plaque number per hippocampus
0
200
400
600
800
NL-G-F
NL-G-F; Lrp6-val
% Coverage of Aβ
0
1
2
3
4
5
NL-G-F
NL-G-F;Lrp6-val
F
Plaque number per hippocampus
0
200
400
600
800
1000
NL-G-F
NL-G-F;Lrp6-val
% Coverageof Aβ
0
2
4
6
NL-G-F
NL-G-F;Lrp6-val

Figure S9

Figure S9. Plaque load is unaltered in *NL-G-F;Lrp6-val* mice. A) Confocal images of Aβ (red) in the hippocampi of *NL-G-F* and *NL-G-F;Lrp6-val* mice at 2 months. Scale bar = 150 µm. **B**) Quantification shows no differences in plaque number were detected. *NL-G-F* N = 5, *NL-G-F;Lrp6-val* N = 5. Unpaired T-test. **C**) Aβ plaques (red) in the hippocampi of *NL-G-F* and *NL-G-F;Lrp6-val* mice at 7 months. Scale bar = 200 µm. **D**) No differences in plaque number or Aβ coverage in the hippocampus were observed. *NL-G-F* N = 8, *NL-G-F;Lrp6-val* N = 6. Unpaired T-test. **E**) Images of Aβ (red) in the hippocampi of *NL-G-F* and *NL-G-F;Lrp6-val* mice at 10 months. Scale bar = 200 µm. **F**) Quantification revealed no differences in plaque number or Aβ coverage. *NL-G-F*: N = 5, *NL-G-F;Lrp6-val*: N = 4. Mann-Whitney tests. Data are represented as mean ± SEM.

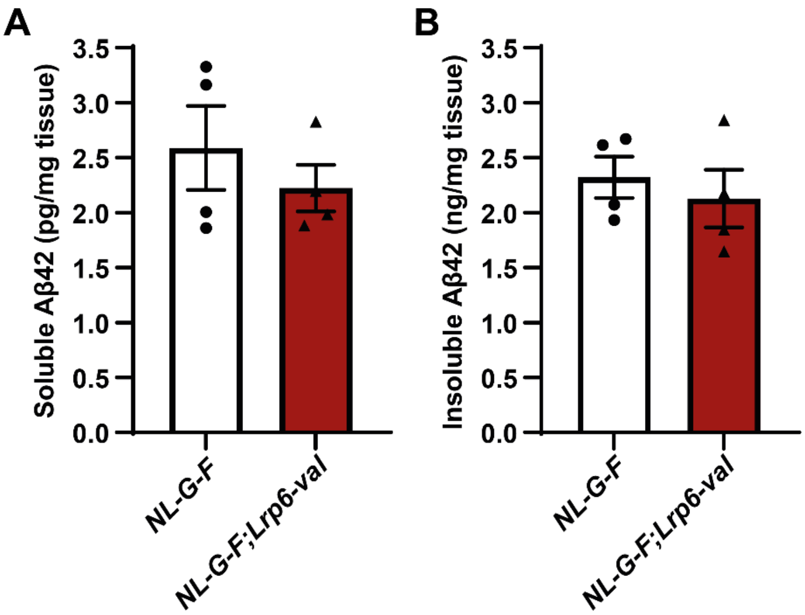

Figure S10

Figure S10. Soluble and insoluble Aβ42 levels are unaffected in *NL-G-F;Lrp6-val* mice. Quantification of soluble (**A**) and insoluble (**B**) Aβ42 by ELISA showed no differences. *NL-G-F* N = 4, *NL-G-F;Lrp6-val* N = 4. Unpaired T-test. Data are represented as mean ± SEM.

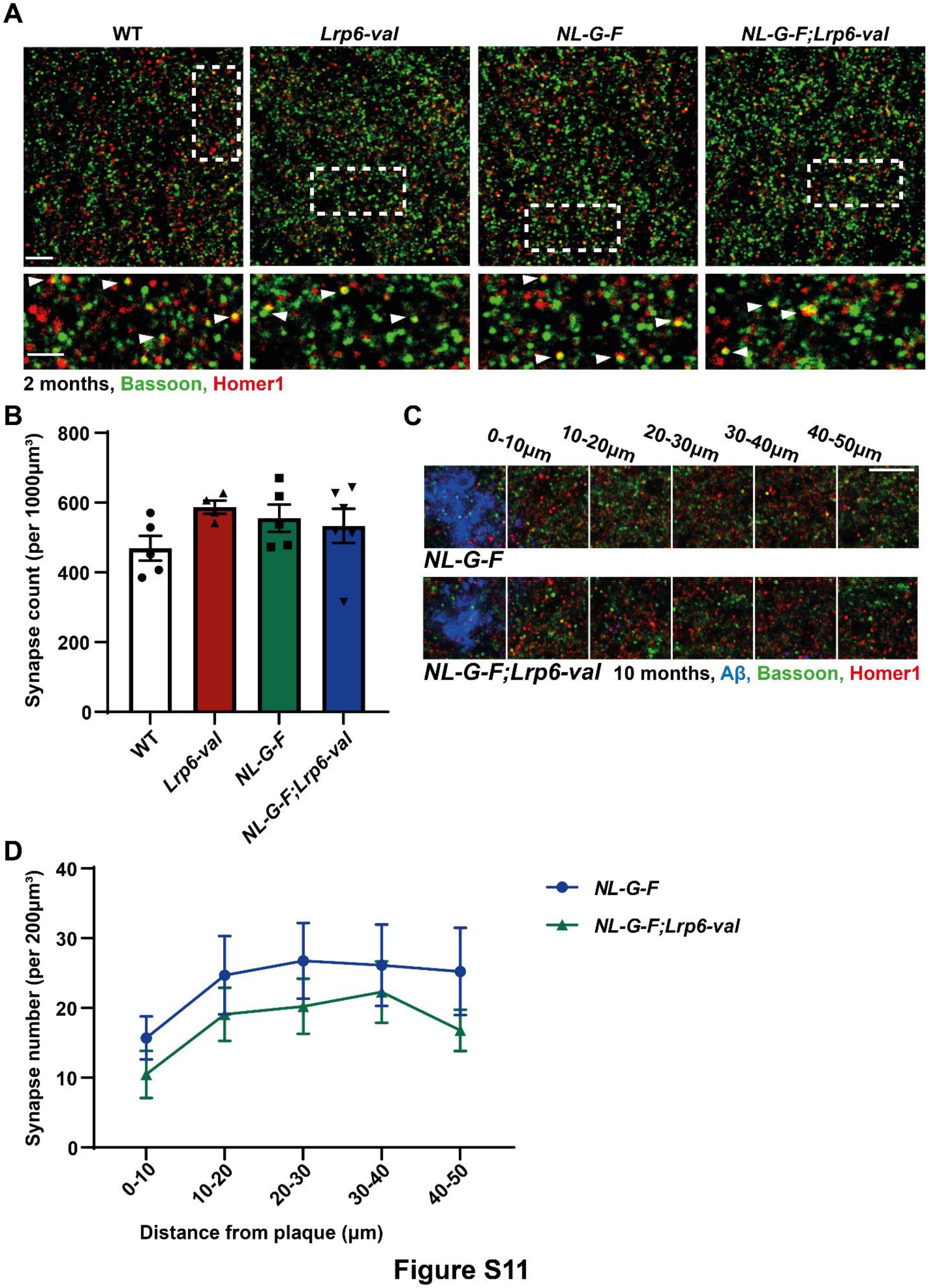
A
WT
Lrp6-val
NL-G-F
NL-G-F;Lrp6-val
2 months, Bassoon, Homer1
B
Synapse count (per 1000μm³)
800
600
400
200
0
WT
Lrp6-val
NL-G-F
NL-G-F;Lrp6-val
C
0-10μm
10-20μm
20-30μm
30-40μm
40-50μm
NL-G-F
NL-G-F;Lrp6-val
10 months, Aβ, Bassoon, Homer1
D
Synapse number (per 200μm³)
40
30
20
10
0
0-10
10-20
20-30
30-40
40-50
Distance from plaque (μm)
NL-G-F
NL-G-F;Lrp6-val

Figure S11

Figure S11. ***Lrp6-val*** **does not affect synapse number in** ***NL-G-F*** **mice at 2 months or 10 months. A**) Confocal images of the CA1 SR of WT, *Lrp6-val*, *NL-G-F* and *NL-G-F;Lrp6-val* mice at 2 months showing Bassoon (green) and Homer1 (red) puncta. Scale bar = 3.8 µm. Insets show higher magnification images of synapses. Scale bar = 2 µm **B**) Quantification of Bassoon and Homer1 puncta and synapse number (co-localised puncta). No differences are detected between any of the different genotypes. WT N = 5, *Lrp6-val* N = 4, *NL-G-F* N = 5, *NL-G-F; Lrp6-val* N = 6. One-way-ANOVA with Tukey's post hoc test. **C**) Confocal images of synapses, co-localised Bassoon (green) and Homer1 (red) puncta, at increasing distances from the centre of an Aβ plaque (blue) in *NL-G-F* and *NL-G-F;Lrp6-val* mice at 10 months. **D**) Quantification revealed no differences in synapse number. Scale bar = 5 µm. *NL-G-F* N = 15 slices from 5 brains, *NL-G-F; Lrp6-val* N = 11 slices from 4 brains. Repeated measure Two-way-ANOVA with Bonferroni's post hoc test. Data are represented as mean ± SEM.

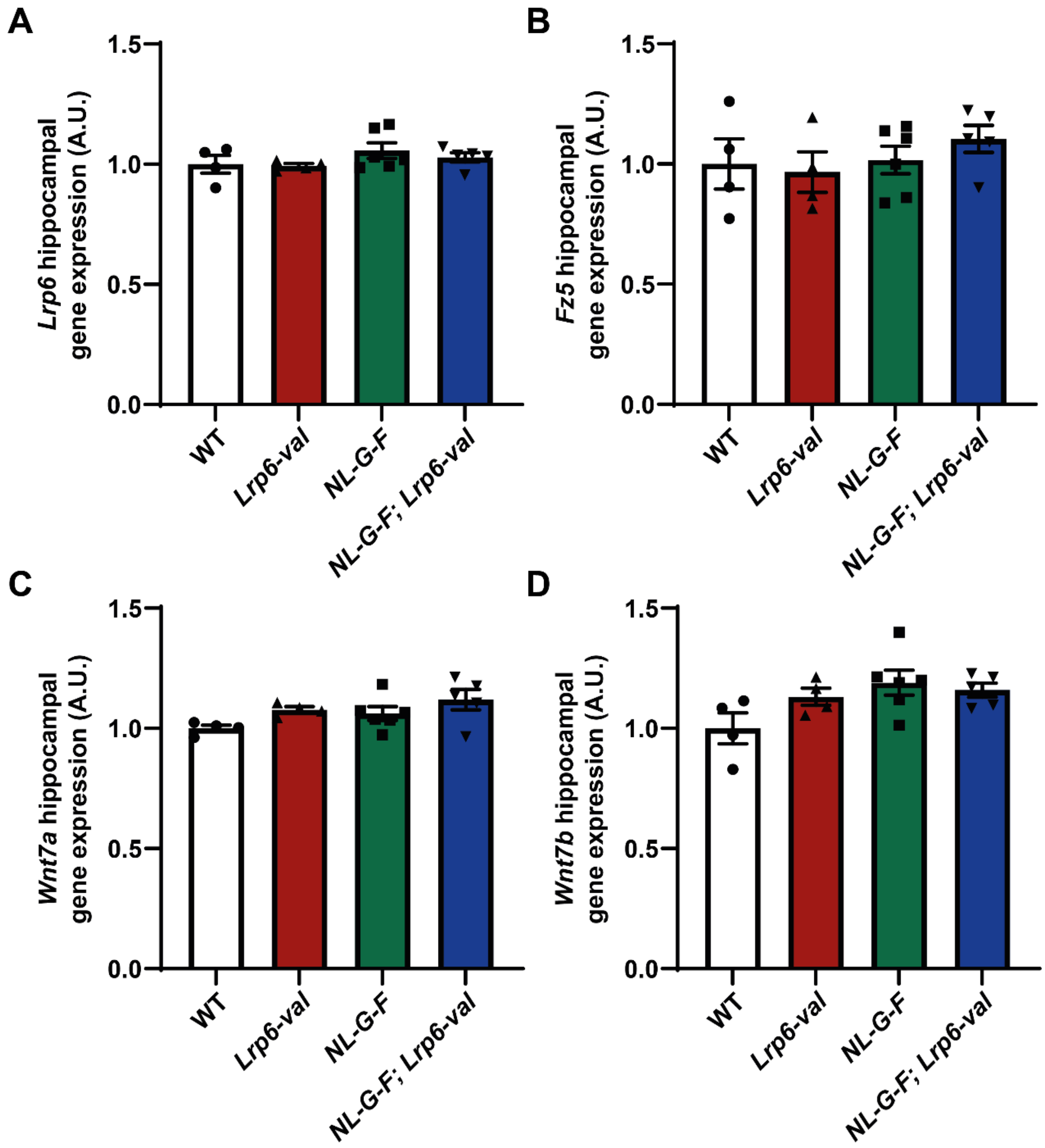

Figure S12

Figure S12. *Lrp6, Fz5, Wnt7a* and *Wnt7b* mRNA levels are unaffected in *Lrp6-val, NL-G-F* and *NL-G-F; Lrp6-val* mice. qPCR analyses of *Lrp6* (**A**), *Fz5* (**B**), *Wnt7a* (**C**) and *Wnt7b* (**D**) expression revealed no differences in the hippocampi of 7-9-month-old WT, *Lrp6-val*, *NL-G-F* and *NL-G-F; Lrp6-val* mice. WT N = 4, *Lrp6-val* N = 4, *NL-G-F* N = 6, *NL-G-F; Lrp6-val* N = 5. One-way-ANOVA with Tukey's post hoc test. Data are represented as mean ± SEM.
